# Efficacy of transcranial direct current stimulation for improving postoperative quality of recovery in elderly patients undergoing lower limb major arthroplasty: a randomized controlled substudy

**DOI:** 10.3389/fneur.2024.1327558

**Published:** 2024-01-24

**Authors:** Mingshu Tao, Jie Yang, Rongguang Liu, Song Zhang, Yuan Han, Chunyan Li, Qi Wei, Dexian Chen, Jiaxing Fang, Hongxing Zhang, Yuwei Wang, He Liu, Jun-Li Cao

**Affiliations:** ^1^Department of Anesthesiology, The Affiliated Hospital of Xuzhou Medical University, Xuzhou, China; ^2^Jiangsu Province Key Laboratory of Anesthesiology and NMPA Key Laboratory for Research and Evaluation of Narcotic and Psychotropic Drugs, Xuzhou Medical University, Xuzhou, China; ^3^Department of Anesthesiology, Renji Hospital School of Medicine, Shanghai Jiao Tong University, Shanghai, China; ^4^Department of Anesthesiology, Eye and ENT Hospital of Fudan University, Shanghai, China; ^5^Department of Intensive Care Medicine, Ng Teng Fong General Hospital, Singapore, Singapore; ^6^Department of Anesthesiology and Clinical Research Center for Anesthesia and Perioperative Medicine, Huzhou Central Hospital, Huzhou, China; ^7^Department of Anesthesiology and Clinical Research Center for Anesthesia and Perioperative Medicine, The Fifth School of Clinical Medicine, Zhejiang Chinese Medical University, Huzhou, China; ^8^Department of Anesthesiology and Clinical Research Center for Anesthesia and Perioperative Medicine, The Affiliated Central Hospital, Huzhou University, Huzhou, China; ^9^Department of Anesthesiology and Clinical Research Center for Anesthesia and Perioperative Medicine, The Affiliated Huzhou Hospital, Zhejiang University School of Medicine, Huzhou, China

**Keywords:** total hip arthroplasty, total knee arthroplasty, transcranial direct current stimulation, quality of recovery, postoperative fatigue, postoperative pain

## Abstract

**Background:**

Previous studies have demonstrated improvements in motor, behavioral, and emotional areas following transcranial direct current stimulation (tDCS), but no published studies have reported the efficacy of tDCS on postoperative recovery quality in patients undergoing lower limb major arthroplasty. We hypothesized that tDCS might improve postoperative recovery quality in elderly patients undergoing lower limb major arthroplasty.

**Methods:**

Ninety-six patients (≥65 years) undergoing total hip arthroplasty (THA) or total knee arthroplasty (TKA) were randomized to receive 2 mA tDCS for 20 min active-tDCS or sham-tDCS. The primary outcome was the 15-item quality of recovery (QoR-15) score on postoperative day one (Т2). Secondary outcomes included the QoR-15 scores at the 2nd hour (T1), the 1st month (Т3), and the 3rd month (Т4) postoperatively, numeric rating scale scores, and fatigue severity scale scores.

**Results:**

Ninety-six elderly patients (mean age, 71 years; 68.7% woman) were analyzed. Higher QoR-15 scores were found in the active-tDCS group at T2 (123.0 [114.3, 127.0] vs. 109.0 [99.3, 115.3]; median difference, 13.0; 95% CI, 8.0 to 17.0; *p* < 0.001). QoR-15 scores in the active-tDCS group were higher at T1 (*p* < 0.001), T3 (*p* = 0.001), and T4 (*p* = 0.001). The pain scores in the active-tDCS group were lower (*p* < 0.001 at motion; *p* < 0.001 at rest). The fatigue degree scores were lower in the active-tDCS group at T1 and T2 (*p* < 0.001 for each).

**Conclusion:**

tDCS may help improve the quality of early recovery in elderly patients undergoing lower limb major arthroplasty.

**Clinical trial registration:**

The trial was registered at the China Clinical Trial Center (ChiCTR2200057777, https://www.chictr.org.cn/showproj.html?proj=162744).

## 1 Introduction

Lower limb major arthroplasty, including total hip arthroplasty (THA) and total knee arthroplasty (TKA), are effective methods for the end-stage treatment of degenerative hip or knee pathologies. There is an increasing trend of THA and TKA each year, with a global incidence of 2.5 million (1) and an incidence of nearly 0.9 million in China in 2019 (2). The lower limb major arthroplasty are very traumatic, especially for older patients. Despite the certain improvements in joint function maintenance and patients’ quality of life by lower limb major arthroplasty, it does carry a risk of significant poor postoperative outcomes, including postoperative pain, fatigue, pulmonary embolism, and death.

Recovery from surgery is a complex process that depends on the patient, surgical and anesthetic factors (3). The primary goal of surgical and anesthetic interventions is to improve patient prognosis, and patients tend to be more concerned with their self-perception of the disease and recovery than objective test data such as laboratory values and imaging (4). However, studies of perioperative interventions usually focus on classical postoperative outcomes, such as morbidity, postoperative organ dysfunction, or postoperative complications, while ignoring patient-centered outcome measures (3). Hence, it is necessary to conduct clinical studies based on patients’ own perceptions to explore the impact of perioperative interventions on the quality of postoperative recovery.

Transcranial direct current stimulation (tDCS) is a non-invasive brain stimulation technique, which could produce a weak electric current on the cerebral cortex, generate subthreshold stimulation of neurons, and regulate the excitability of cortical neurons (5). It not only affects the cortical activity under electrodes but also modulates the functional activity of brain networks (6). It has been evidenced that tDCS could enhance the endogenous pain control system and elevate the pain threshold by activating brain regions (7). In addition, previous studies have demonstrated modifications in motor, behavioral, and emotional areas benefitting from tDCS (8–10). Therefore, we would like to explore whether tDCS could improve the quality of recovery for elderly patients undergoing lower limb major arthroplasty.

## 2 Methods

### 2.1 Ethics approval and registration

The study protocol was approved by the Ethics Committee of the Affiliated Hospital of Xuzhou Medical University (XYFY2022-KL001-01; date: 25 January 2022). The trial was registered before patient enrollment at the China Clinical Trial Center (ChiCTR2200057777; date: 17 March 2022). Written informed consent was obtained from all subjects participating in the trial or a legal surrogate. The study was conducted in the Department of Anesthesiology and the Department of Orthopedic Surgery at the Affiliated Hospital of Xuzhou Medical University from March to August 2022.

This study was predefined as a substudy of our original study before patient enrollment began. The original study was a prospective, single-center, randomized, double-blind, controlled clinical trial that assessed the efficacy of tDCS on the incidence of postoperative delirium (POD) in elderly patients undergoing lower limb major arthroplasty (11). Both studies employed the same intervention, albeit with distinct objectives and varying sample sizes. Considering that the intervention in this study (using tDCS) had not been used in perioperative patients and to reduce the impact of the intervention on patients, when both protocols were submitted to the ethics committee of the hospital, the ethics committee of the affiliated hospital permitted to use the same patients for the trial, subject to Consolidated Standards of Reporting Trials guidelines.

### 2.2 Study participants

All patients enrolled in the trial received detailed information about the study protocol. Inclusion criteria were ≥65 years old; scheduled to undergo THA or TKA under elective general anesthesia of intravenous and inhalation; American Society of Anesthesiologists rating grade II or III. Exclusion criteria were patient refusal to sign the consent form; neuropsychiatric disorders and previous history of neurological or psychiatric disorders; cranial or scalp injury; visual and hearing impairment and difficulty communicating; history of severe cardiovascular disease; metal implants in the body (e.g., cardiac implants); substance or alcohol abuse; mini-mental state examination score < 15; severe liver or kidney dysfunction. In addition, participants would be eliminated for the following reasons: voluntary withdrawal or poor compliance; violation of the protocol; use of other drugs or methods that affected the trial’s outcome indicators; and failure of the follow-up.

### 2.3 Randomization and blinding

Participants were centrally randomized using a computer-generated number table and were randomized in a 1:1 ratio to either active-tDCS or sham-tDCS. The grouping information was placed in sealed opaque envelopes by an investigator. The envelope would not be opened until the patient was transferred to the postanesthesia care unit. The investigators who collected or processed data and participants were blinded to the group assignment. After the completion of data collection for both studies, the trial results were subjected to statistical analysis by the researchers who subsequently were unblinded.

### 2.4 Anesthesia procedures

After entering the operating room, the patients received the venous catheter placement, accompanied by the monitor on electrocardiogram, oxygen saturation, and invasive arterial blood pressure. Midazolam 0.05 mg kg^−1^, etomidate 0.3 mg kg^−1^, sufentanil 0.5 ug kg^−1^, and rocuronium bromide 1 mg kg^−1^ were intravenously injected for anesthesia induction. The femoral nerve block was used under ultrasound guidance with 20 mL of 0.5% ropivacaine after the patient had been anesthetized. Anesthesia maintenance: continuous intravenous infusion of propofol 4–6 mg kg^−1^ h^−1^, remifentanil 0.1–0.3 ug kg^−1^ min^−1^, and continuous inhalation of 1% sevoflurane to maintain BIS values between 40 and 60. The end-expiratory carbon dioxide partial pressure was maintained between 35 and 45 mmHg. Vasoactive drugs were given as necessary to maintain heart rate and blood pressure fluctuations within 20% of baseline. Sevoflurane was stopped 30 min before the end of surgery. The surgical approaches for THA and TKA were the direct lateral approach (12) and the mini-medial parapatellar approach (13), respectively. After surgery, all patients were transferred to the postanesthesia care unit and were given atropine 0.5 mg, neostigmine 1 mg, and flumazenil 0.5 mg. The drug of the patient’s self-control analgesic pump was: sufentanil 1.5 ug kg^−1^, tropisetron 6 mg and saline to 100 mL, continuous infusion rate of 2 mL h^−1^, self-control analgesic dose of 0.5 mL, locked time of 15 min. The patient’s self-control analgesic pump lasted approximately 2 days. When participants’ postoperative pain score exceeded 4, additional analgesic medication was preferentially administered instead of compression analgesic pumps, with analgesic use determined by the surgeon on the ward.

### 2.5 tDCS intervention

**Figure 1:**
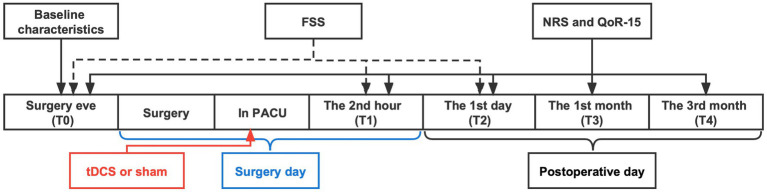
Timeline of the experimental design of the two experimental sessions (active-tDCS and sham-tDCS). tDCS, transcranial direct current stimulation; FSS, fatigue severity scale; NRS, numeric rating scale; QoR-15, 15-item quality of recovery; PACU, postanesthesia care unit.

When patients were conscious, muscle tone returned to normal, breathing was uniform, and the ideal tidal volume was achieved, the tracheal catheter could be removed. All patients received one session of active-tDCS or sham-tDCS after catheter removal. The electrodes were both soaked with saline (NaCl 0.9%) and fixed by the stretchy hat. The anode was placed over the left dorsolateral prefrontal cortex (DLPFC), and the cathode was placed over the right orbitofrontal area. The placement of electrodes and the utilization of instrumentation are detailed in Supplementary Figure A.1. For the active-tDCS group, a constant current of 2 mA was applied for 20 min with a 30-s ramp-up phase at the beginning and a 30-s ramp-down phase at the end. For the sham-tDCS group, the patients only received a 30-s ramp-up phase at the beginning and a 30-s ramp-down phase at the end. The patient’s local skin and vital signs were closely monitored during the use of tDCS by researchers who implemented the intervention. The stimulation was stopped promptly for any abnormalities occurrence, which was reported to the supervising physician for punctual intervention. The timeline of the experimental design of the two experimental sessions is shown in Figure 1.

### 2.6 Outcomes

The primary outcome was the quality of recovery assessed by the 15-item quality of recovery (QoR-15) score on the 1st day (Т2) after surgery. The QoR-15 scale involving 15 questions was employed to measure five domains of the postoperative health status of patients, including physical comfort, emotional state, physical independence, psychological support, and pain (14). Each item is scored from 0 to 10, with a total score of 150 points, as higher scores represented better recovery.

Secondary outcomes included QoR-15 scores at the 2nd hour (T1), the 1st month (Т3), and the 3rd month (Т4) after surgery; numeric rating scale (NRS) scores at T1-T4; fatigue severity scale (FSS) scores at T1 and T2. The NRS score is 0 to 10, with 0 for no pain, 1 to 3 for mild pain, 4 to 6 for moderate pain, and 7 to 10 for severe pain (15). The FSS is a 9-item scale with a score from 0 to 7 for each entry and a total score of 63 (16).

The post-hoc analysis included nausea and vomiting, the time of the first postoperative analgesic use, and hospital length of stay. In addition, we hierarchically assessed POD during the first 3 postoperative days, followed by the QoR-15 score on Т2, and the comparison was stratified according to the type of surgery. The effect of tDCS on postoperative recovery quality, pain scores, and fatigue severity scores were also assessed separately according to the type of surgery. The outcomes were assessed directly by the investigators on the ward or by telephone from patients who had been discharged from the hospital.

### 2.7 Statistical analysis

Perioperative interventions resulting in a change of 6 points for the QoR-15 signify the minimal clinically important difference (MCID) (17). The standard deviation was 9.8 according to the preliminary experiment. The test power was 0.80, and the allowable error was 0.05. The size of each group was calculated to be 43, and 48 patients were required in each group after factoring in a 10% drop-out rate.

All analyses were conducted with the intention-to-treat principle, and missing data were largely assumed to be missing at random. There were no missing data for the primary outcome, and missing data for all secondary outcomes were less than 5%, we did not perform an imputation of missing data. For the primary outcome, we used the Manne-Whitney U test and median difference as effect size. For the secondary outcomes collected at multiple time points, we built the generalized estimated equation model. The generalized estimated equation model was applied with repeated measurement data as the dependent variable, and the treatment, time, and treatment multiplied by time interaction as independent variables. The treatment-by-time interaction term was tested first. If significant, between-group differences at each time point were tested. If not significant, the treatment’s main effect was tested next. Otherwise, the *p*-value threshold for statistical significance was calculated using the Bonferroni correction to adjust for multiple comparisons in secondary outcomes conducted. The Chi-square test or the Fisher exact test was performed for categorical variables expressed as frequency. Assessment for normal distribution was performed with the Shapiro–Wilk test. Normally distributed data are presented as mean and standard deviation and compared by the unpaired t-test. and non-normally distributed data as median and interquartile range and compared by the Manne-Whitney U test. The median difference was calculated by the Hodges-Lehmann estimate based on the Mann–Whitney U test. The cumulative incidence of the first postoperative analgesic use was analyzed by Kaplan–Meier survival analyses and the difference in incidence between groups was compared by the Log-rank test.

We hierarchically assessed POD during the first 3 postoperative days followed by the QoR-15 score on Т2 using the concept of the win ratio. The win ratio was motivated by the Finkelstein–Schoenfeld test, with the aim of providing an estimate and confidence interval (CI) for the treatment effect (win rate), as well as a *p*-value (18, 19). The method gives higher importance to POD. The pairwise comparison proceeded hierarchically, using POD, followed by the QoR-15 score when patients could not be differentiated on the POD.

To address the robustness of the intention-to-treat analysis (especially concerning the issue of missing data due to dropout and protocol breach), the per-protocol analysis was used as sensitivity analysis. Statistical analysis was conducted on SPSS 26.0 and R version 4.2.2, with 2-sided and *p* < 0.05 considered statistically significant.

## 3 Results

### 3.1 Study patients

**Figure 2:**
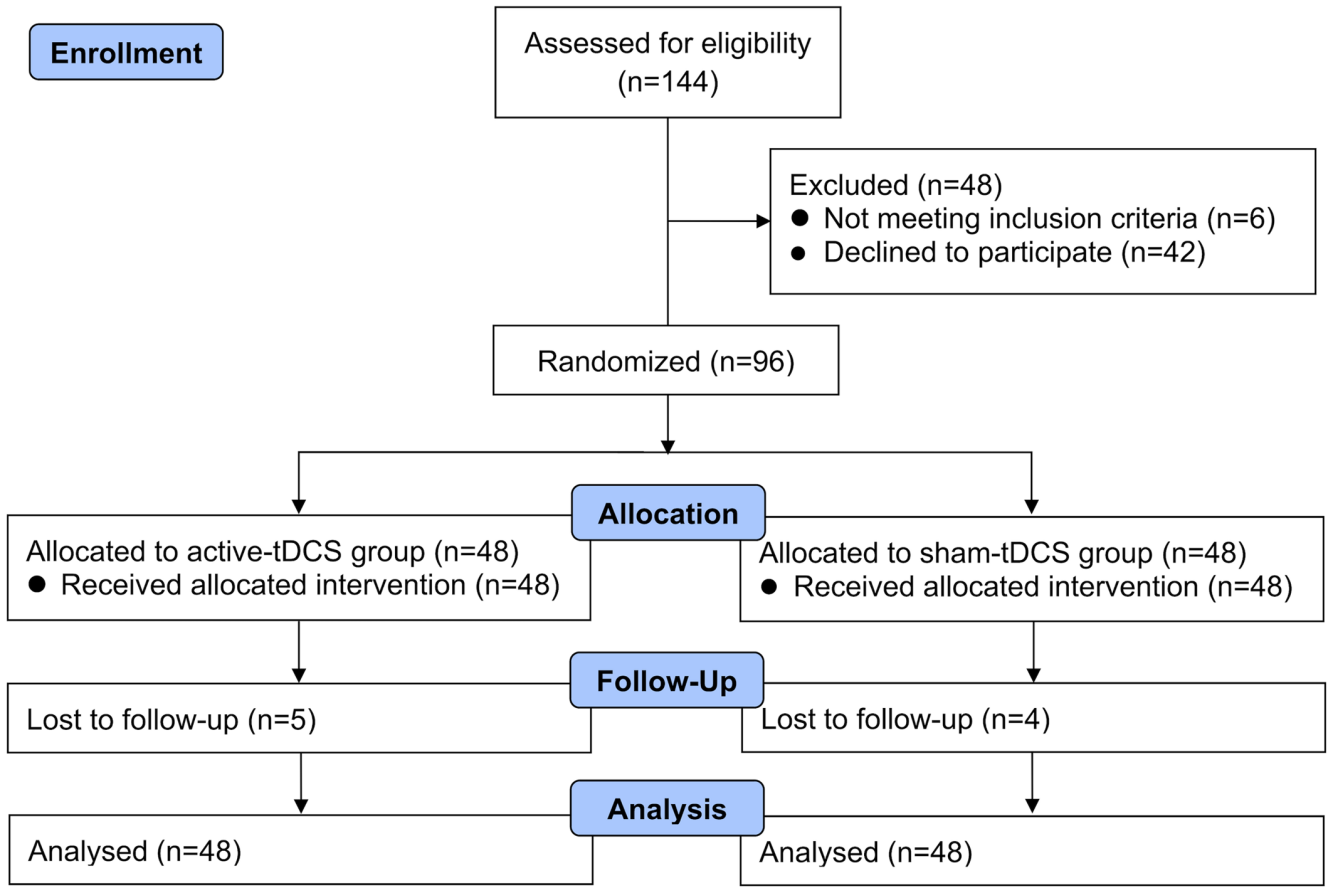
Consolidated standards of reporting trials flow study diagram describing patients’ enrolment, allocation, and analysis procedure through the study. tDCS, transcranial direct current stimulation.

A total of 144 patients were initially assessed as eligible, and 96 patients were randomized into two groups after excluding 48 patients. Nine patients failed to complete the trial due to loss to follow-up after discharge from the hospital (refusal of outcome assessment after discharge, *n* = 3; wrong phone number for follow-up visits, *n* = 6). Finally, 87 patients completed the trial. The Consolidated Standards of Reporting Trials flow diagram for this trial was shown in Figure 2. Data of all patients were analyzed according to their assigned group. Demographic and clinical characteristics at baseline between groups were detailed in Table 1.

**Table 1:** Baseline characteristics of study participants.

	Active-tDCS (*n* = 48)	Sham-tDCS (*n* = 48)	*P*-value
Age (year)	70.0 (4.9)	71.0 (5.5)	0.223
Gender			0.186
Male	18 (37.5%)	12 (25.0%)	
Female	30 (62.5%)	36 (75.0%)	
Weight (kg)	65.2 (9.6)	64.3 (12.5)	0.697
Height (cm)	161.3 (6.6)	160.6 (7.7)	0.654
BMI (kg m^−2^)	25.0 (3.2)	24.8 (3.7)	0.723
ASA			0.066
II	28 (58.3%)	19 (39.6%)	
III	20 (41.7%)	29 (60.4%)	
Type of operation			0.832
TKA	31 (64.6%)	30 (62.5%)	
THA	17 (35.4%)	18 (37.5%)	
Etiology			0.913
Osteoarthritis	32	30	
Femoral neck fracture	8	9	
Aseptic necrosis of the femoral head	8	9	
Treatment received prior to enrollment			0.307
Conservative treatment	26	21	
None	22	27	
Age-adjusted Charlson Comorbidity Index	3.0 (2.0, 4.0)	3.0 (3.0, 4.0)	0.182
FRAIL			0.087
Robust	2 (4.2%)	8 (16.7%)	
Prefrail	17 (35.4%)	11 (22.9%)	
Frail	29 (60.4%)	29 (60.4%)	
Quality of Recovery-15 score	139.0 (134.5, 143.0)	139.0 (131.3, 143.0)	0.460
Numeric Rating Scale score at motion	3.0 (1.0, 4.0)	3.0 (1.0, 4.0)	0.929
Numeric Rating Scale score at rest	1.0 (0.0, 2.0)	1.5 (0.0, 2.8)	0.716
Fatigue severity scale score	53.0 (47.3, 55.0)	54.0 (48.0, 57.8)	0.175
Duration of surgery (min)	100.0 (80.0, 127.5)	95.0 (81.3, 110.0)	0.458
Duration of anesthesia (min)	125.0 (101.3, 147.5)	120.0 (105.0, 140.0)	0.620
Estimated blood loss (ml)	100.0 (50.0, 142.5)	100.0 (50.0, 187.5)	0.918
Femoral nerve block			0.100
Yes	31 (64.6%)	23 (47.9%)	
No	17 (35.4%)	25 (52.0%)	

Data are presented as mean (standard deviation), n (%), or median (interquartile range). tDCS, transcranial direct current stimulation; BMI, body mass index; ASA, American Society of Anesthesiologists rating; THA, total hip arthroplasty; TKA, total knee arthroplasty; FRAIL, Fatigue, Resistance, Ambulation, Illness and Loss of Weight Scale. Conservative treatment includes taking anti-inflammatory and analgesic medications, acupuncture and physical therapy.

### 3.2 Primary outcomes

**Figure 3:**
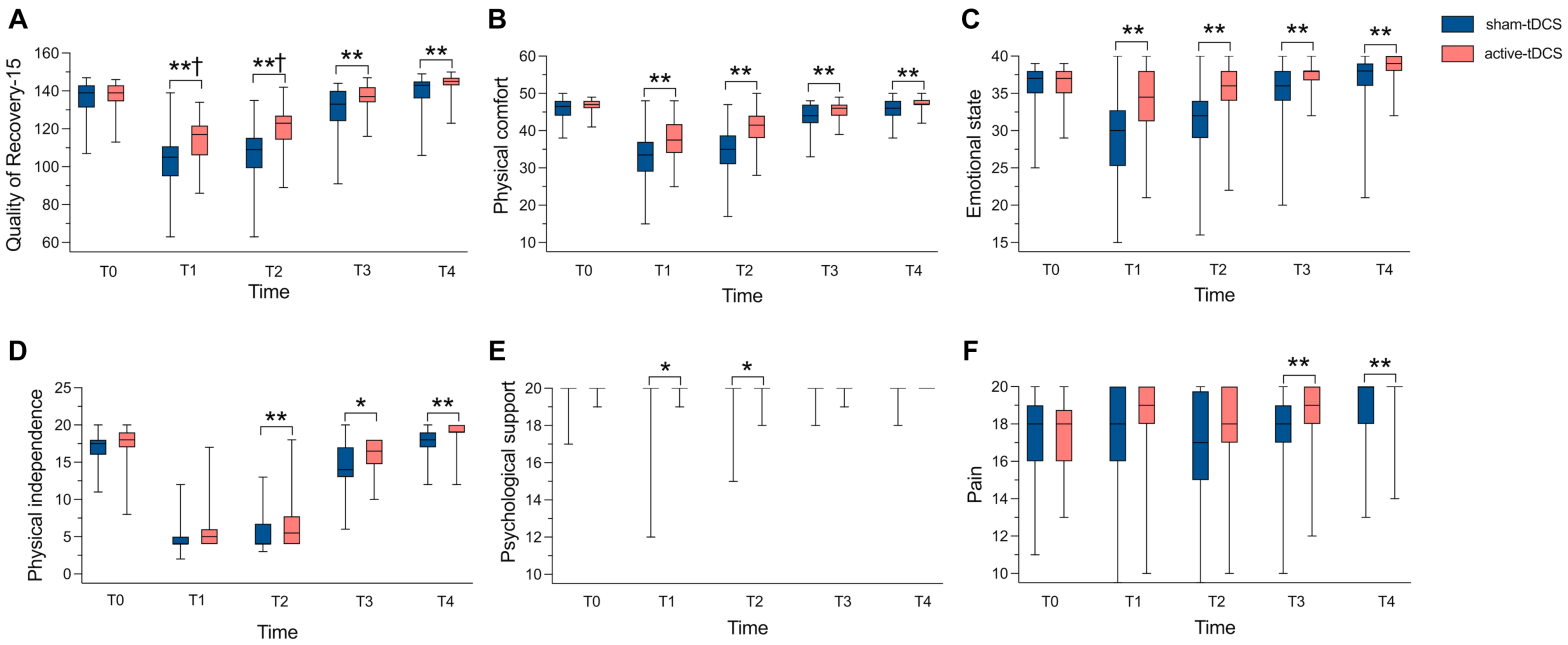
Comparison of QoR-15 scores and its 5 dimensions between groups. (A) Comparison of QoR-15 scores; (B) Comparison of Physical comfort scores; (C) Comparison of Emotional state scores; (D) Comparison of Physical independence scores; (E) Comparison of Psychological support scores; (F) Comparison of Pain scores. Median values shown as solid line within box of 25th and 75th percentile values. Whiskers represent maximum and minimum values. QoR-15, 15-item quality of recovery; tDCS, transcranial direct current stimulation. ^*^p < 0.05. ^**^p < 0.01. ^†^Median difference > 6.

The QoR-15 scores at T2 were 123.0 (114.3, 127.0) and 109.0 (99.3, 115.3) for the active-tDCS group and the sham-tDCS group (*p* < 0.001), with a median difference of 13.0 (95% CI, 8.0 to 17.0; Figure 3).

### 3.3 Secondary outcomes

The QoR-15 scores were significantly higher in the active-tDCS group at T1 compared to the sham-tDCS group (*p* < 0.001), median (interquartile range): 117.0 (106.0, 121.8) vs. 105.0 (95.0, 110.8), with median difference of 12.0 (95% CI, 7.0 to 16.0). The median difference of the QoR-15 score between groups at T3 and T4 were 4 and 3, respectively (Figure 3; Supplementary Table A.1).

No interaction was found between time and group for the NRS scores at motion (*p* = 0.077) or at rest (*p* = 0.101). The treatment main effect was revealed significant between groups (*p* < 0.001 at motion; *p* < 0.001 at rest; Table 2; Supplementary Figure A.2). Supplementary Figure A.3 indicated the comparison of pain degrees at different times between the active-tDCS and sham-tDCS groups. The interaction between time and group for the FSS scores was revealed (*p* < 0.001). Compared to the sham-tDCS group, the FSS scores in the active-tDCS group were significantly decreased at T1 (*p* < 0.001) and T2 (*p* < 0.001; Table 2).

**Table 2:** Secondary outcomes.

	Active-tDCS (*n* = 48)	Sham-tDCS (*n* = 48)	Difference (95% CI)^†^	*P*-value
**Numerical rating scale at motion**				
Treatment-by-time interaction				0.077^*^
Main effect				<0.001
T1	1.0 (1.0, 1.0)	1.0 (1.0, 4.0)	−1.0 (−1.0, 0.0)	0.001
T2	2.0 (1.0, 4.0)	4.0 (1.0, 5.0)	−1.0 (−2.0, 0.0)	0.004
T3	1.0 (1.0, 3.0)	2.0 (1.0, 4.0)	−1.0 (−1.0, 0.0)	0.009
T4	0.0 (0.0, 1.0)	1.0 (0.0, 2.0)	0.0 (−1.0, 0.0)	0.014
**Numerical rating scale at rest**				
Treatment-by-time interaction				0.101^*^
Main effect				<0.001
T1	0.0 (0.0, 0.0)	0.0 (0.0, 3.0)	0.0 (−1.0, 0.0)	0.002
T2	1.0 (0.0, 2.8)	3.0 (0.0, 3.0)	−1.0 (−2.0, 0.0)	0.007
T3	1.0 (0.0, 2.0)	1.0 (1.0, 3.0)	−1.0 (−1.0, 0.0)	0.019
T4	0.0 (0.0, 0.0)	0.0 (0.0, 2.0)	0.0 (0.0, 0.0)	0.031
**Fatigue severity scale**				
Treatment-by-time interaction				
Main effect				<0.001
T1	49.0 (45.0, 54.0)	56.0 (53.0, 61.8)	−7.0 (−9.0, −4.0)	<0.001
T2	46.0 (44.3, 54.0)	57.5 (50.8, 63.0)	−9.0 (−11.0, −6.0)	<0.001
Nausea and vomiting	11 (22.9%)	15 (31.3%)	−8.4% (−26.0, 9.2%)	0.358
Hospital length of stay (d)	10.0 (8.0, 12.0)	9.0 (8.0, 13.0)	0.0 (−1.0, 1.0)	0.527

Data are presented as *n* (%) or median (interquartile range). tDCS, transcranial direct current stimulation; T1, the 2nd hour postoperatively; T2, the 1st day postoperatively; T3, the 1st month postoperatively; T4, the 3rd month postoperatively; CI, confidence interval. ^*^Because no treatment-by-time interaction was found, no Bonferroni correction was made for assessing treatment effect at each time point. ^†^active-tDCS—sham-tDCS.

### 3.4 *Post hoc* outcomes

**Figure 4:**
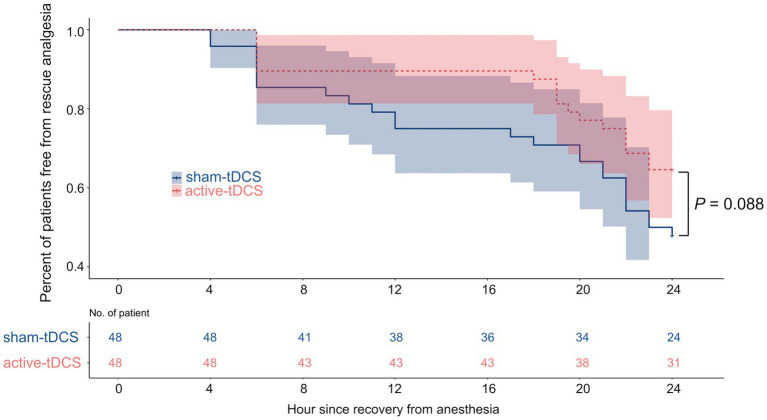
Kaplan–Meier survival plot of time to first rescue analgesia. tDCS, transcranial direct current stimulation.

No significant difference was found in the time to the first rescue analgesia after surgery (*p* = 0.088; Figure 4), nausea and vomiting, and hospital length of stay between groups (Table 2). During the study period, no complications were observed among patients who completed the treatment protocol with considerable tolerance on the tDCS.

There was no statistical difference in the incidence of POD between the active-tDCS group (2 [4.2%]) and sham-tDCS group (7 [14.6%]; relative risk, 0.29; 95% CI, 0.06 to 1.31; *p* = 0.161). The active-tDCS group was superior to the sham-tDCS group in the analysis that hierarchically assessed POD during the first 3 postoperative days followed by the QoR-15 score on Т2 (*p* < 0.001), and the win ratio was 3.13 (95% CI, 1.83 to 5.36; Supplementary Table A.2). Across prespecified subgroups, including those based on the type of surgery, the difference in POD and QoR-15 score favored active-tDCS over sham-tDCS (Supplementary Table A.2).

Compared to the sham-tDCS group, the QoR-15 scores were significantly higher among patients undergoing THA and TKA in the active-tDCS group at T2 (*p* < 0.001 and *p* = 0.003 respectively; Supplementary Tables A.3, A.4). The NRS scores were slightly altered when stratified by the type of surgery(Supplementary Tables A.3, A.4). No significant difference in the NRS scores at motion or rest was found at any time point between groups among patients undergoing THA (Supplementary Table A.3).

The baseline characteristics of study participants in the per-protocol analysis are shown in Supplementary Table A.5. In the per-protocol analysis, the QoR-15 scores at T2 were 123.0 (114.0, 127.0) and 109.0 (101.3, 116.8) for the active-tDCS group and the sham-tDCS group (*p* < 0.001), with a median difference of 12.0 (95% CI, 6.0 to 16.0; Supplementary Table A.6; Supplementary Figure A.4). The results of the per-protocol analysis were generally consistent with the intention-to-treat analysis results (Supplementary Tables A.5–A.8; Supplementary Figures A.4–A.7). The detailed results from the generalized estimated equation model in the interaction model and the model without time interaction are shown in the appendix (Supplementary Tables A.9–A.20).

## 4 Discussion

This study investigated the effect of tDCS on postoperative quality of recovery in elderly patients undergoing lower limb major arthroplasty, including THA or TKA. We have found that tDCS may help improve the quality of early recovery in elderly patients undergoing lower limb major arthroplasty.

The recovery from surgery depends on the patient, surgical and anesthetic factors (3). Patients tend to be more concerned with their self-perception of the disease and recovery than objective test data such as laboratory values and imaging (4). Hence, the quality of recovery was selected as the primary outcome of this study. Compared to other quality of recovery scales, the QoR-15 scale may be a more straightforward, practical, and convenient scale (20). Moreover, the QoR-15 scale is suitable for patients who need to be followed up by telephone for weeks or months after surgery in clinical trials (21). The present study demonstrated significantly higher QoR-15 scores at the 2nd hour and the 1st day after surgery in the active-tDCS group, with a median difference of 12 and 13 respectively, exceeding the MCID.

In this substudy, the active-tDCS group was found to be statistically unable to reduce the incidence of POD compared to the sham-tDCS group. In the original study, the incidence of POD during the first postoperative 3 days was significantly lower in the active-tDCS group (3 [4.9%] of 61 patients) than that in the sham-tDCS group (12 [19.7%] of 61 patients; relative risk, 0.250; 95% CI, 0.074 to 0.842; *p* = 0.013) (11). This difference in results may be due to the fact that the sample size of this substudy was too small to meet the predefined sample size for assessing the outcome of POD. However, the hierarchical composite of POD and QoR-15 score using the win ratio in this substudy found the result to be statistically significant. The win ratio can provide greater statistical power to detect and quantify a treatment difference by using all available information contained in the component outcomes (22). The Finkelstein–Schoenfeld method is a validated technique that increases the sensitivity and power of the analysis of smaller cohorts (23) and prioritizes the importance of POD. There were slight differences between the NRS score and the pain dimension score of QoR-15. This was most likely because the pain dimension score focuses more on pain frequency while NRS emphasizes pain severity.

The study found that 44% of TKA patients and 27% of THA patients could develop mild CPSP 3–4 years after surgery, and 15% of TKA patients and 6% of THA patients had very severe CPSP (24). The best way to prevent CPSP is to alleviate acute pain by early intervention, inhibit the persistence and progression of acute pain, and prevent peripheral sensitization and central sensitization. It was found that the effect of tDCS-related analgesia became obvious 12 h postoperatively after only 1 or 2 sessions (25). Hence, using tDCS before the onset of pain may enhance the endogenous pain modulation system and elevate the pain threshold. Despite the statistically lower pain scores in the active-tDCS group in this trial, it still failed to reach the MCID (26). The unsatisfactory analgesic effect may be related to only one session of tDCS utilized and the more traumatic of THA than TKA. Therefore, multiple sessions of tDCS are required to achieve the MCID in pain and enhance the endogenous pain modulation system.

Postoperative fatigue plays an important role in postoperative recovery and delays the return to regular activity after surgery (27). It may be one of the main complaints of patients and may last longer than pain, affecting their quality of life and preventing a return to normal function and activities (28). Postoperative pain is the main risk factor for early postoperative fatigue (29). In addition, advanced age, surgical trauma, decreased nutritional status, anxiety, depression, and inflammatory reaction are risk factors for postoperative fatigue. tDCS is known to reduce patients’ pain and inflammatory factor levels and alleviate anxiety and depression (27, 30, 31). These may be the reasons why this trial found that tDCS reduced the fatigue level of patients.

This study has the following limitations: First, this trial was a single-center study and focused on elderly patients undergoing lower limb major arthroplasty. The findings may be less generalizable to elderly patients with other conditions. Second, only a single session was given in this study without multiple sessions. Multiple sessions are required to enhance the analgesic effect of tDCS. Third, this trial was only followed up to 3 months postoperatively by telephone. Future studies are still needed to explore the long-term effects of tDCS. Fourth, the study measured outcomes using subjective assessment scales and did not use objective indicators such as imaging techniques and biological markers. However, the results of this study were reliable as the follow-up interviewers were trained and blinded to the experimental groups.

## 5 Conclusion

Under the conditions of the present study, a single anodal tDCS session on the left DLPFC may help improve the quality of early recovery in elderly patients undergoing lower limb major arthroplasty. The data suggest that this neuromodulatory approach may be part of the effective interventions available for postoperative recovery. This study may expand the application of electrical stimulation in the field of perioperative and postoperative rehabilitation, and provide a good foundation for future research on neuromodulatory approach.

## Data availability statement

The datasets presented in this article are not readily available because there are stringent restrictions in making the research dataset of the clinical studies publicly available. Requests to access the datasets should be directed to J-LC, caojl0310@aliyun.com.

## Ethics statement

The studies involving humans were approved by Ethics Committee of the Affiliated Hospital of Xuzhou Medical University. The studies were conducted in accordance with the local legislation and institutional requirements. The participants provided their written informed consent to participate in this study.

## Author contributions

MT: Conceptualization, Data curation, Formal analysis, Validation, Writing – original draft, Writing – review & editing. JY: Conceptualization, Data curation, Investigation, Methodology, Writing – review & editing. RL: Conceptualization, Data curation, Investigation, Methodology, Writing – review & editing. SZ: Conceptualization, Data curation, Funding acquisition, Project administration, Writing – review & editing. YH: Funding acquisition, Supervision, Writing – review & editing. CL: Investigation, Writing – review & editing. QW: Investigation, Writing – review & editing. DC: Investigation, Writing – review & editing. JF: Investigation, Writing – review & editing. HZ: Funding acquisition, Validation, Writing – review & editing. YW: Conceptualization, Methodology, Writing – review & editing. HL: Conceptualization, Formal analysis, Funding acquisition, Methodology, Project administration, Supervision, Writing – original draft, Writing – review & editing. J-LC: Conceptualization, Funding acquisition, Project administration, Resources, Supervision, Writing – review & editing.

## References

[1] Nemeth B Nelissen R Arya R Cannegieter S. Preventing VTE following total hip and knee arthroplasty: is prediction the future? J Thromb Haemost. (2021) 19:41–5. doi: 10.1111/jth.15132, PMID: 33043553 PMC7839761

[2] Feng B Zhu W Bian YY Chang X Cheng KY Weng XS. China artificial joint annual data report. Chin Med J. (2020) 134:752–3. doi: 10.1097/CM9.0000000000001196, PMID: 33725711 PMC7990009

[3] Lee L Tran T Mayo NE Carli F Feldman LS. What does it really mean to “recover” from an operation? Surgery. (2014) 155:211–6. doi: 10.1016/j.surg.2013.10.002, PMID: 24331759

[4] Bowyer A Royse C. Approaches to the measurement of post-operative recovery. Best Pract Res Clin Anaesthesiol. (2018) 32:269–76. doi: 10.1016/j.bpa.2018.02.001, PMID: 30522717

[5] Camacho-Conde JA Gonzalez-Bermudez MDR Carretero-Rey M Khan ZU. Brain stimulation: a therapeutic approach for the treatment of neurological disorders. CNS Neurosci Ther. (2022) 28:5–18. doi: 10.1111/cns.13769, PMID: 34859593 PMC8673710

[6] Kim K Sherwood MS McIntire LK McKinley RA Ranganath C. Transcranial direct current stimulation modulates connectivity of left dorsolateral prefrontal cortex with distributed cortical networks. J Cogn Neurosci. (2021) 33:1381–95. doi: 10.1162/jocn_a_01725, PMID: 34496406

[7] Fregni F Macedo IC Spezia-Adachi LN Scarabelot VL Laste G Souza A. Transcranial direct current stimulation (tDCS) prevents chronic stress-induced hyperalgesia in rats. Brain Stimul. (2018) 11:299–301. doi: 10.1016/j.brs.2017.11.009, PMID: 29175003

[8] Schretlen DJ van Steenburgh JJ Varvaris M Vannorsdall TD Andrejczuk MA Gordon B. Can transcranial direct current stimulation improve cognitive functioning in adults with schizophrenia? Clin Schizophr Relat Psychoses. (2017) 11:133–42. doi: 10.3371/CSRP.SCST.103114 25367166

[9] Brunoni AR Boggio PS De Raedt R Benseñor IM Lotufo PA Namur V. Cognitive control therapy and transcranial direct current stimulation for depression: a randomized, double-blinded, controlled trial. J Affect Disord. (2014) 162:43–9. doi: 10.1016/j.jad.2014.03.026 24767004

[10] Park SH Seo JH Kim YH Ko MH. Long-term effects of transcranial direct current stimulation combined with computer-assisted cognitive training in healthy older adults. NeuroReport. (2014) 25:122–6. doi: 10.1097/WNR.0000000000000080, PMID: 24176927

[11] Tao M Zhang S Han Y Li C Wei Q Chen D. Efficacy of transcranial direct current stimulation on postoperative delirium in elderly patients undergoing lower limb major arthroplasty: a randomized controlled trial. Brain Stimul. (2023) 16:88–96. doi: 10.1016/j.brs.2023.01.839, PMID: 36682718

[12] Petis S Howard JL Lanting BL Vasarhelyi EM. Surgical approach in primary total hip arthroplasty: anatomy, technique and clinical outcomes. Can J Surg. (2015) 58:128–39. doi: 10.1503/cjs.007214, PMID: 25799249 PMC4373995

[13] Scuderi GR Tenholder M Capeci C. Surgical approaches in mini-incision total knee arthroplasty. Clin Orthop Relat Res. (2004) 428:61–7. doi: 10.1097/01.blo.0000148574.79874.d0, PMID: 15534520

[14] Stark PA Myles PS Burke JA. Development and psychometric evaluation of a postoperative quality of recovery score: the QoR-15. Anesthesiology. (2013) 118:1332–40. doi: 10.1097/ALN.0b013e318289b84b, PMID: 23411725

[15] Hjermstad MJ Fayers PM Haugen DF Caraceni A Hanks GW Loge JH. Studies comparing numerical rating scales, verbal rating scales, and visual analogue scales for assessment of pain intensity in adults: a systematic literature review. J Pain Symptom Manag. (2011) 41:1073–93. doi: 10.1016/j.jpainsymman.2010.08.016, PMID: 21621130

[16] Krupp LB LaRocca NG Muir-Nash J Steinberg AD. The fatigue severity scale. Application to patients with multiple sclerosis and systemic lupus erythematosus. Arch Neurol. (1989) 46:1121–3. doi: 10.1001/archneur.1989.00520460115022 2803071

[17] Myles PS Myles DB. An updated minimal clinically important difference for the QoR-15 scale. Anesthesiology. (2021) 135:934–5. doi: 10.1097/ALN.0000000000003977, PMID: 34543410

[18] Pocock SJ Ariti CA Collier TJ Wang D. The win ratio: a new approach to the analysis of composite endpoints in clinical trials based on clinical priorities. Eur Heart J. (2012) 33:176–82. doi: 10.1093/eurheartj/ehr352, PMID: 21900289

[19] Finkelstein DM Schoenfeld DA. Combining mortality and longitudinal measures in clinical trials. Stat Med. (1999) 18:1341–54. doi: 10.1002/(sici)1097-0258(19990615)18:11<1341::aid-sim129>3.0.co;2-7 10399200

[20] Wessels E Perrie H Scribante J Jooma Z. Quality of recovery in the perioperative setting: a narrative review. J Clin Anesth. (2022) 78:110685. doi: 10.1016/j.jclinane.2022.110685, PMID: 35190342

[21] Myles PS Shulman MA Reilly J Kasza J Romero L. Measurement of quality of recovery after surgery using the 15-item quality of recovery scale: a systematic review and meta-analysis. Br J Anaesth. (2022) 128:1029–39. doi: 10.1016/j.bja.2022.03.009 35430086

[22] Redfors B Gregson J Crowley A McAndrew T Ben-Yehuda O Stone GW. The win ratio approach for composite endpoints: practical guidance based on previous experience. Eur Heart J. (2020) 41:4391–9. doi: 10.1093/eurheartj/ehaa665, PMID: 32901285

[23] Maurer MS Schwartz JH Gundapaneni B Elliott PM Merlini G Waddington-Cruz M. Tafamidis treatment for patients with transthyretin amyloid cardiomyopathy. N Engl J Med. (2018) 379:1007–16. doi: 10.1056/NEJMoa1805689 30145929

[24] Wylde V Hewlett S Learmonth ID Dieppe P. Persistent pain after joint replacement: prevalence, sensory qualities, and postoperative determinants. Pain. (2011) 152:566–72. doi: 10.1016/j.pain.2010.11.023, PMID: 21239114

[25] Borckardt JJ Reeves ST Milliken C Carter B Epperson TI Gunselman RJ. Prefrontal versus motor cortex transcranial direct current stimulation (tDCS) effects on post-surgical opioid use. Brain Stimul. (2017) 10:1096–101. doi: 10.1016/j.brs.2017.09.006 28917592 PMC5675751

[26] Bahreini M Safaie A Mirfazaelian H Jalili M. How much change in pain score does really matter to patients? Am J Emerg Med. (2020) 38:1641–6. doi: 10.1016/j.ajem.2019.158489, PMID: 31744654

[27] Zargar-Shoshtari K Hill AG. Postoperative fatigue: a review. World J Surg. (2009) 33:738–45. doi: 10.1007/s00268-008-9906-0 19189174

[28] Mendy N Moriceau J Sacuto Y Besnier E Clavier T Ndangang MM. Postoperative fatigue after day surgery: prevalence and risk factors. A prospective observational study. Minerva Anestesiol. (2020) 86:1269–76. doi: 10.23736/S0375-9393.20.14358-X, PMID: 32755085

[29] Lefaucheur JP Chalah MA Mhalla A Palm U Ayache SS Mylius V. The treatment of fatigue by non-invasive brain stimulation. Neurophysiol Clin. (2017) 47:173–84. doi: 10.1016/j.neucli.2017.03.003 28410876

[30] Amidfar M Ko YH Kim YK. Neuromodulation and cognitive control of emotion. Adv Exp Med Biol. (2019) 1192:545–64. doi: 10.1007/978-981-32-9721-0_27 31705513

[31] Pacheco-Barrios K Cardenas-Rojas A Thibaut A Costa B Ferreira I Caumo W. Methods and strategies of tDCS for the treatment of pain: current status and future directions. Expert Rev Med Devices. (2020) 17:879–98. doi: 10.1080/17434440.2020.1816168, PMID: 32845195 PMC7674241

