# Supplementary material for: Efficacy of transcranial direct current stimulation for improving postoperative quality of recovery in elderly patients undergoing lower limb major arthroplasty: a randomized controlled substudy
Source: Front Neurol. 2024 Jan 24;15:1327558. doi: 10.3389/fneur.2024.1327558 (PMC10849132; doi:10.3389/fneur.2024.1327558)
Supplement: Supplementary file 1 [file Data_Sheet_1.docx]

**Supplementary Materials**

**Table A.1.** Comparison of Quality of Recovery-15 at multiple points in time between groups.

|  | active-tDCS (n=48) | sham-tDCS (n=48) | Median difference (95% CI)† | *P*-value |
| --- | --- | --- | --- | --- |
| **Quality of Recovery-15** |  |  |  |  |
| Treatment-by-time interaction |  |  |  | <0.001 |
| Main effect |  |  |  | <0.001 |
| T0 | 139.0 (134.5, 143.0) | 139.0 (131.3, 143.0) | 1.0 (-2.0, 4.0) | 0.276 |
| T1 | 117.0 (106.0, 121.8) | 105.0 (95.0, 110.8) | 12.0 (7.0, 16.0) | <0.001 |
| T2 | 123.0 (114.3, 127.0) | 109.0 (99.3, 115.3) | 13.0 (8.0, 17.0) | <0.001 |
| T3 | 137.0 (134.0, 142.0) | 133.0 (124.0, 140.0) | 4.0 (1.0, 8.0) | 0.001 |
| T4 | 145.0 (143.0, 147.0) | 143.0 (136.0, 145.0) | 3.0 (1.0, 5.0) | 0.001 |
| **Physical comfort** |  |  |  |  |
| Treatment-by-time interaction |  |  |  | 0.001 |
| Main effect |  |  |  | <0.001 |
| T0 | 47.0 (46.0, 48.0) | 46.5 (44.0, 48.0) | 0.0 (0.0, 1.0) | 0.187 |
| T1 | 37.5 (34.0, 41.8) | 33.5 (29.0, 37.0) | 4.0 (2.0, 7.0) | 0.001 |
| T2 | 41.5 (38.0, 44.0) | 35.0 (31.0, 38.8) | 6.0 (4.0, 8.0) | <0.001 |
| T3 | 46.0 (44.0, 47.0) | 44.0 (42.0, 47.0) | 1.0 (0.0, 3.0) | 0.002 |
| T4 | 47.0 (47.0, 48.3) | 46.0 (44.0, 48.0) | 1.0 (0.0, 2.0) | 0.003 |
| **Emotional state** |  |  |  |  |
| Treatment-by-time interaction |  |  |  | <0.001 |
| Main effect |  |  |  | <0.001 |
| T0 | 37.0 (35.0, 38.0) | 37.0 (35.0, 38.0) | 0.0 (-1.0, 1.0) | 0.546 |
| T1 | 34.5 (31.3, 38.0) | 30.0 (25.3, 32.8) | 5.0 (3.0, 7.0) | <0.001 |
| T2 | 36.0 (34.0, 38.0) | 32.0 (29.0, 34.0) | 4.0 (3.0, 6.0) | <0.001 |
| T3 | 38.0 (36.8, 38.0) | 36.0 (34.0, 38.0) | 1.0 (0.0, 2.0) | 0.001 |
| T4 | 39.0 (38.0, 40.0) | 38.0 (36.0, 39.0) | 1.0 (1.0, 2.0) | 0.001 |
| **Physical independence** |  |  |  |  |
| Treatment-by-time interaction |  |  |  | 0.386* |
| Main effect |  |  |  | 0.002 |
| T0 | 18.0 (17.0, 19.0) | 17.5 (16.0, 18.0) | 1.0 (0.0, 1.0) | 0.047 |
| T1 | 5.0 (4.0, 6.0) | 4.0 (4.0, 5.0) | 0.0 (0.0, 1.0) | 0.062 |
| T2 | 5.5 (4.0, 7.8) | 4.0 (4.0, 6.8) | 1.0 (0.0, 1.0) | 0.008 |
| T3 | 16.5 (14.8, 18.0) | 14.0 (13.0, 17.0) | 1.0 (0.0, 2.0) | 0.020 |
| T4 | 19.0 (19.0, 20.0) | 18.0 (17.0, 19.0) | 1.0 (0.0, 2.0) | <0.001 |
| **Psychological support** |  |  |  |  |
| Treatment-by-time interaction |  |  |  | 0.017 |
| Main effect |  |  |  | 0.008 |
| T0 | 20.0 (20.0, 20.0) | 20.0 (20.0, 20.0) | 0.0 (0.0, 0.0) | 0.007 |
| T1 | 20.0 (20.0, 20.0) | 20.0 (20.0, 20.0) | 0.0 (0.0, 0.0) | 0.011 |
| T2 | 20.0 (20.0, 20.0) | 20.0 (20.0, 20.0) | 0.0 (0.0, 0.0) | 0.030 |
| T3 | 20.0 (20.0, 20.0) | 20.0 (20.0, 20.0) | 0.0 (0.0, 0.0) | 0.922 |
| T4 | 20.0 (20.0, 20.0) | 20.0 (20.0, 20.0) | 0.0 (0.0, 0.0) | 0.427 |
| **Pain** |  |  |  |  |
| Treatment-by-time interaction |  |  |  | 0.057* |
| Main effect |  |  |  | 0.005 |
| T0 | 18.0 (16.0, 18.8) | 18.0 (16.0, 19.0) | 0.0 (-1.0, 0.0) | 0.697 |
| T1 | 19.0 (18.0, 20.0) | 18.0 (16.0, 20.0) | 0.0 (0.0, 1.0) | 0.173 |
| T2 | 18.0 (17.0, 20.0) | 17.0 (15.0, 19.8) | 1.0 (0.0, 2.0) | 0.055 |
| T3 | 19.0 (18.0, 20.0) | 18.0 (17.0, 19.0) | 1.0 (0.0, 1.0) | <0.001 |
| T4 | 20.0 (20.0, 20.0) | 20.0 (18.0, 20.0) | 0.0 (0.0, 1.0) | <0.001 |

Data are presented as median (interquartile range). tDCS, transcranial direct current stimulation; T0, the day before surgery; T1, the 2nd hour postoperatively; T2, the 1st day postoperatively; T3, the 1st month postoperatively; T4, the 3rd month postoperatively; CI, confidence interval.

*: Because no Treatment-by-time interaction was found, no Bonferroni correction was made for assessing treatment effect at each time point. †: active-tDCS - sham-tDCS.**Table A.2.** Overall and subgroup results as calculated with the use of the Finkelstein–Schoenfeld method, Postoperative delirium, and Quality of Recovery-15 score.

|  | No. of Patients | Postoperative delirium† | Quality of Recovery-15 score on T2† | No. of “winners” | No. of “losers” | **Win Ratio (95%CI)** | ***P*-value*** |
| --- | --- | --- | --- | --- | --- | --- | --- |
| **Overall** |  |  |  | 1722 | 550 | 3.13 (1.83, 5.36) | <0.001 |
| active-tDCS | 48 | 2 (4.2%) | 123.0 (114.3, 127.0) |  |  |  |  |
| sham-tDCS | 48 | 7 (14.6%) | 109.0 (99.3, 115.3) |  |  |  |  |
| **Type of operation** |  |  |  |  |  |  |  |
| **THA** |  |  |  | 262 | 44 | 5.95 (1.97, 17.97) | 0.002 |
| active-tDCS | 17 | 1 (5.9%) | 123.0 (115.5, 127.5) |  |  |  |  |
| sham-tDCS | 18 | 5 (27.8%) | 109.0 (100.5, 111.0) |  |  |  |  |
| **TKA** |  |  |  | 639 | 274 | 2.33 (1.23, 4.41) | 0.009 |
| active-tDCS | 31 | 1 (3.2%) | 123.0 (111.0, 127.0) |  |  |  |  |
| sham-tDCS | 30 | 2 (6.7%) | 108.5 (98.8, 121.3) |  |  |  |  |

tDCS, transcranial direct current stimulation; THA, total hip arthroplasty; TKA, total knee arthroplasty; T2, the 1st day postoperatively; CI, confidence interval.

*: *P*-value from Finkelstein-Schoenfeld method. †: Data are presented as n (%) and median (interquartile range).**Table A.3.** Comparison of Quality of Recovery-15, Numerical rating scale, and Fatigue severity scale scores between groups in total hip arthroplasty.

|  | active-tDCS (n=17) | sham-tDCS (n=18) | Median difference (95% CI)† | *P*-value |
| --- | --- | --- | --- | --- |
| **Quality of Recovery-15** |  |  |  |  |
| Treatment-by-time interaction |  |  |  | 0.001 |
| Main effect |  |  |  | 0.001 |
| T0 | 140.0 (129.5, 143.0) | 139.0 (132.8, 143.0) | 0.0 (-4.0, 6.0) | 0.813 |
| T1 | 116.0 (105.0, 118.0) | 100.0 (92.0, 106.0) | 13.0 (8.0, 21.0) | 0.001 |
| T2 | 123.0 (115.5, 127.5) | 109.0 (100.5, 111.0) | 15.0 (9.0, 22.0) | <0.001 |
| T3 | 140.5 (135.5, 144.5) | 135.0 (123.5, 138.5) | 7.0 (1.0, 14.0) | 0.013 |
| T4 | 146.0 (144.3, 148.0) | 143.0 (133.5, 145.5) | 4.0 (1.0, 10.0) | 0.072 |
| **Physical comfort** |  |  |  |  |
| Treatment-by-time interaction |  |  |  | 0.001 |
| Main effect |  |  |  | <0.001 |
| T0 | 47.0 (45.0, 48.0) | 46.0 (44.8, 47.3) | 1.0 (-1.0, 2.0) | 0.592 |
| T1 | 37.0 (31.0, 40.5) | 30.5 (26.5, 33.3) | 6.0 (2.0, 10.0) | 0.003 |
| T2 | 41.0 (38.0, 44.5) | 33.0 (30.8, 36.5) | 7.0 (4.0, 11.0) | <0.001 |
| T3 | 47.0 (45.0, 47.0) | 44.0 (40.0, 47.0) | 2.0 (0.0, 5.0) | 0.008 |
| T4 | 47.0 (46.3, 49.0) | 47.0 (43.0, 48.5) | 1.5 (-1.0, 4.0) | 0.075 |
| **Emotional state** |  |  |  |  |
| Treatment-by-time interaction |  |  |  | 0.001 |
| Main effect |  |  |  | 0.003 |
| T0 | 37.0 (33.5, 38.0) | 37.5 (35.0, 38.3) | 0.0 (-2.0, 1.0) | 0.447 |
| T1 | 34.0 (30.0, 37.5) | 30.0 (25.5, 32.0) | 5.0 (2.0, 8.0) | 0.003 |
| T2 | 36.0 (34.0, 38.0) | 32.0 (29.8, 33.3) | 5.0 (2.0, 7.0) | 0.001 |
| T3 | 38.0 (37.3, 39.0) | 37.0 (33.5, 38.0) | 2.0 (0.0, 3.0) | 0.019 |
| T4 | 40.0 (38.0, 40.0) | 38.0 (36.0, 39.0) | 1.5 (1.0, 2.0) | 0.102 |
| **Physical independence** |  |  |  |  |
| Treatment-by-time interaction |  |  |  | 0.002 |
| Main effect |  |  |  | 0.041 |
| T0 | 18.0 (16.5, 19.0) | 17.0 (16.0, 18.3) | 1.0 (-1.0, 2.0) | 0.959 |
| T1 | 4.0 (4.0, 5.0) | 4.0 (4.0, 4.0) | 0.0 (0.0, 1.0) | 0.862 |
| T2 | 5.0 (4.0, 6.0) | 4.0 (4.0, 4.3) | 1.0 (0.0, 2.0) | 0.014 |
| T3 | 17.0 (15.3, 18.0) | 14.0 (11.5, 16.5) | 3.0 (0.0, 4.0) | 0.025 |
| T4 | 19.0 (19.0, 20.0) | 18.0 (16.5, 19.0) | 1.0 (0.0, 2.0) | 0.114 |
| **Psychological support** |  |  |  |  |
| Treatment-by-time interaction |  |  |  | 0.286* |
| Main effect |  |  |  | 0.108 |
| T0 | 20.0 (20.0, 20.0) | 20.0 (20.0, 20.0) | 0.0 (0.0, 0.0) | 0.782 |
| T1 | 20.0 (20.0, 20.0) | 20.0 (19.8, 20.0) | 0.0 (0.0, 0.0) | 0.386 |
| T2 | 20.0 (20.0, 20.0) | 20.0 (20.0, 20.0) | 0.0 (0.0, 0.0) | 0.568 |
| T3 | 20.0 (20.0, 20.0) | 20.0 (20.0, 20.0) | 0.0 (0.0, 0.0) | 0.763 |
| T4 | 20.0 (20.0, 20.0) | 20.0 (20.0, 20.0) | 0.0 (0.0, 0.0) | 1.000 |
| **Pain** |  |  |  |  |
| Treatment-by-time interaction |  |  |  | 0.328* |
| Main effect |  |  |  | 0.451 |
| T0 | 18.0 (16.0, 18.5) | 18.0 (16.8, 19.0) | 0.0 (-1.0, 1.0) | 0.525 |
| T1 | 18.0 (17.5, 20.0) | 18.5 (16.8, 20.0) | 0.0 (-1.0, 1.0) | 0.782 |
| T2 | 19.0 (18.0, 20.0) | 18.5 (15.0, 20.0) | 0.0 (-1.0, 2.0) | 0.386 |
| T3 | 19.0 (18.3, 20.0) | 19.0 (17.5, 19.0) | 1.0 (0.0, 1.0) | 0.058 |
| T4 | 20.0 (20.0, 20.0) | 19.0 (18.0, 20.0) | 0.0 (0.0, 1.0) | 0.118 |
| **Numerical rating scale at motion** |  |  |  |  |
| Treatment-by-time interaction |  |  |  | 0.461* |
| Main effect |  |  |  | 0.253 |
| T0 | 2.0 (1.0, 4.0) | 2.0 (1.0, 4.0) | 0.0 (0.0, 2.0) | 0.463 |
| T1 | 1.0 (1.0, 3.0) | 1.0 (1.0, 4.3) | 0.0 (-1.0, 0.0) | 0.483 |
| T2 | 1.0 (1.0, 3.0) | 1.0 (1.0, 4.0) | 0.0 (-1.0, 0.0) | 0.245 |
| T3 | 1.0 (0.0, 2.8) | 2.0 (1.0, 4.0) | -1.0 (-2.0, 0.0) | 0.118 |
| T4 | 0.0 (0.0, 1.0) | 1.0 (0.0, 2.0) | 0.0 (-1.0, 0.0) | 0.231 |
| **Numerical rating scale at rest** |  |  |  |  |
| Treatment-by-time interaction |  |  |  | 0.548* |
| Main effect |  |  |  | 0.377 |
| T0 | 2.0 (0.0, 2.5) | 0.5 (0.0, 2.0) | 0.0 (0.0, 2.0) | 0.546 |
| T1 | 0.0 (0.0, 1.0) | 0.0 (0.0, 3.0) | 0.0 (-1.0, 0.0) | 0.546 |
| T2 | 0.0 (0.0, 1.5) | 0.5 (0.0, 3.0) | 0.0 (-1.0, 0.0) | 0.443 |
| T3 | 1.0 (0.0, 2.8) | 1.0 (0.0, 3.0) | 0.0 (-1.0, 0.0) | 0.345 |
| T4 | 0.0 (0.0, 1.0) | 0.0 (0.0, 1.5) | 0.0 (0.0, 0.0) | 0.606 |
| **Fatigue severity scale** |  |  |  |  |
| Treatment-by-time interaction |  |  |  | 0.001 |
| Main effect |  |  |  | <0.001 |
| T0 | 53.0 (44.5, 56.0) | 52.0 (43.5, 55.0) | 0.0 (-5.0, 6.0) | 0.820 |
| T1 | 48.0 (46.0, 54.0) | 56.5 (53.5, 60.8) | -7.0 (-11.0, -2.0) | 0.001 |
| T2 | 45.0 (41.5, 54.0) | 58.0 (54.5, 63.0) | -11.5 (-16.0, -7.0) | <0.001 |

Data are presented as median (interquartile range). tDCS, transcranial direct current stimulation; T0, the day before surgery; T1, the 2nd hour postoperatively; T2, the 1st day postoperatively; T3, the 1st month postoperatively; T4, the 3rd month postoperatively; CI, confidence interval.

*: Because no treatment-by-time interaction was found, no Bonferroni correction was made for assessing treatment effect at each time point. †: active-tDCS－sham-tDCS.**Table A.4.** Comparison of Quality of Recovery-15, Numerical rating scale, and Fatigue severity scale scores between groups in total knee arthroplasty.

|  | active-tDCS (n=31) | sham-tDCS (n=30) | Median difference (95% CI)† | *P*-value |
| --- | --- | --- | --- | --- |
| **Quality of Recovery-15** |  |  |  |  |
| Treatment-by-time interaction |  |  |  | 0.079* |
| Main effect |  |  |  | <0.001 |
| T0 | 138.5 (136.0, 143.0) | 138.5 (130.5, 143.0) | 1.0 (-2.0, 5.0) | 0.483 |
| T1 | 118.0 (109.0, 124.0) | 108.0 (101.0, 112.0) | 10.0 (4.0, 16.0) | 0.002 |
| T2 | 123.0 (111.0, 127.0) | 108.5 (98.8, 121.3) | 11.0 (4.0, 18.0) | 0.003 |
| T3 | 137.0 (134.0, 138.8) | 132.5 (125.5, 140.0) | 3.0 (-1.0, 7.0) | 0.102 |
| T4 | 144.0 (142.0, 147.0) | 142.5 (136.0, 145.0) | 3.0 (1.0, 5.0) | 0.008 |
| **Physical comfort** |  |  |  |  |
| Treatment-by-time interaction |  |  |  | 0.196* |
| Main effect |  |  |  | 0.003 |
| T0 | 47.0 (46.0, 48.0) | 47.0 (43.8, 48.0) | 0.0 (-1.0, 1.0) | 0.686 |
| T1 | 38.0 (34.0, 43.0) | 35.5 (29.0, 40.0) | 3.0 (-1.0, 7.0) | 0.112 |
| T2 | 42.0 (37.0, 44.0) | 35.5 (31.0, 42.0) | 5.0 (1.0, 8.0) | 0.012 |
| T3 | 46.0 (43.8, 46.0) | 44.0 (42.0, 46.0) | 1.0 (0.0, 2.0) | 0.144 |
| T4 | 47.0 (47.0, 48.0) | 46.0 (44.0, 47.3) | 1.0 (0.0, 2.0) | 0.033 |
| **Emotional state** |  |  |  |  |
| Treatment-by-time interaction |  |  |  | 0.013 |
| Main effect |  |  |  | <0.001 |
| T0 | 37.0 (35.0, 38.0) | 37.0 (33.8, 38.3) | 0.0 (-1.0, 1.0) | 0.200 |
| T1 | 35.0 (32.0, 38.0) | 30.0 (24.8, 34.0) | 4.0 (2.0, 7.0) | 0.001 |
| T2 | 36.0 (34.0, 38.0) | 32.0 (28.0, 35.3) | 4.0 (2.0, 6.0) | 0.001 |
| T3 | 37.5 (36.0, 38.0) | 36.0 (33.8, 38.0) | 1.0 (0.0, 2.0) | 0.016 |
| T4 | 39.0 (38.0, 40.0) | 38.0 (36.8, 38.3) | 1.0 (0.0, 2.0) | 0.005 |
| **Physical independence** |  |  |  |  |
| Treatment-by-time interaction |  |  |  | 0.974* |
| Main effect |  |  |  | 0.016 |
| T0 | 18.0 (17.0, 19.0) | 18.0 (16.0, 18.3) | 1.0 (0.0, 2.0) | 0.081 |
| T1 | 5.0 (4.0, 6.0) | 4.0 (4.0, 5.0) | 0.0 (0.0, 1.0) | 0.164 |
| T2 | 6.0 (4.0, 8.0) | 4.0 (4.0, 8.0) | 0.0 (0.0, 2.0) | 0.157 |
| T3 | 16.0 (13.8, 18.0) | 15.0 (13.0, 17.0) | 1.0 (0.0, 2.0) | 0.216 |
| T4 | 19.0 (18.8, 20.0) | 18.0 (17.0, 19.0) | 1.0 (0.0, 2.0) | 0.008 |
| **Psychological support** |  |  |  |  |
| Treatment-by-time interaction |  |  |  | 0.095* |
| Main effect |  |  |  | 0.036 |
| T0 | 20.0 (20.0, 20.0) | 20.0 (19.0, 20.0) | 0.0 (0.0, 0.0) | 0.010 |
| T1 | 20.0 (20.0, 20.0) | 20.0 (20.0, 20.0) | 0.0 (0.0, 0.0) | 0.206 |
| T2 | 20.0 (20.0, 20.0) | 20.0 (20.0, 20.0) | 0.0 (0.0, 0.0) | 0.145 |
| T3 | 20.0 (20.0, 20.0) | 20.0 (20.0, 20.0) | 0.0 (0.0, 0.0) | 0.317 |
| T4 | 20.0 (20.0, 20.0) | 20.0 (20.0, 20.0) | 0.0 (0.0, 0.0) | 0.317 |
| **Pain** |  |  |  |  |
| Treatment-by-time interaction |  |  |  | 0.155* |
| Main effect |  |  |  | 0.002 |
| T0 | 18.0 (16.0, 19.0) | 17.0 (16.0, 19.0) | 0.0 (-1.0, 1.0) | 0.988 |
| T1 | 19.0 (18.0, 20.0) | 18.0 (15.0, 19.3) | 1.0 (0.0, 2.0) | 0.049 |
| T2 | 18.0 (17.0, 19.0) | 16.5 (15.0, 19.3) | 1.0 (0.0, 2.0) | 0.047 |
| T3 | 19.0 (18.0, 19.3) | 18.0 (16.8, 19.0) | 1.0 (0.0, 2.0) | 0.002 |
| T4 | 20.0 (20.0, 20.0) | 20.0 (18.8, 20.0) | 0.0 (0.0, 1.0) | 0.001 |
| **Numerical rating scale at motion** |  |  |  |  |
| Treatment-by-time interaction |  |  |  | 0.169* |
| Main effect |  |  |  | <0.001 |
| T0 | 3.0 (1.0, 4.0) | 4.0 (1.0, 4.0) | 0.0 (-1.0, 0.0) | 0.465 |
| T1 | 1.0 (0.0, 1.0) | 1.5 (1.0, 4.0) | -1.0 (-3.0, 0.0) | 0.001 |
| T2 | 3.0 (1.0, 4.0) | 4.0 (3.8, 5.0) | -1.0 (-2.0, 0.0) | 0.004 |
| T3 | 1.0 (1.0, 3.3) | 2.5 (1.0, 5.0) | 0.0 (-2.0, 0.0) | 0.031 |
| T4 | 0.0 (0.0, 1.0) | 1.0 (0.0, 2.5) | 0.0 (-1.0, 0.0) | 0.044 |
| **Numerical rating scale at rest** |  |  |  |  |
| Treatment-by-time interaction |  |  |  | 0.220* |
| Main effect |  |  |  | <0.001 |
| T0 | 1.0 (0.0, 2.0) | 2.0 (0.0, 3.0) | 0.0 (-1.0, 0.0) | 0.327 |
| T1 | 0.0 (0.0, 0.0) | 1.0 (0.0, 3.0) | 0.0 (-2.0, 0.0) | 0.001 |
| T2 | 2.0 (0.0, 3.0) | 3.0 (2.0, 4.0) | -1.0 (-2.0, 0.0) | 0.005 |
| T3 | 1.0 (0.0, 2.0) | 1.5 (1.0, 3.3) | -1.0 (-1.0, 0.0) | 0.029 |
| T4 | 0.0 (0.0, 0.0) | 0.0 (0.0, 2.0) | 0.0 (0.0, 0.0) | 0.025 |
| **Fatigue severity scale** |  |  |  |  |
| Treatment-by-time interaction |  |  |  | 0.021 |
| Main effect |  |  |  | 0.001 |
| T0 | 53.0 (48.0, 55.0) | 55.0 (50.8, 60.0) | -3.0 (-6.0, 0.0) | 0.073 |
| T1 | 49.0 (45.0, 54.0) | 55.0 (52.3, 62.3) | -8.0 (-10.0, -3.0) | <0.001 |
| T2 | 47.0 (45.0, 54.0) | 56.5 (46.0, 63.0) | -8.0 (-10.0, -2.0) | 0.004 |

Data are presented as median (interquartile range). tDCS, transcranial direct current stimulation; T0, the day before surgery; T1, the 2nd hour postoperatively; T2, the 1st day postoperatively; T3, the 1st month postoperatively; T4, the 3rd month postoperatively; CI, confidence interval.

*: Because no treatment-by-time interaction was found, no Bonferroni correction was made for assessing treatment effect at each time point. †: active-tDCS－sham-tDCS.**Table A.5.** Baseline characteristics of study participants in per-protocol analysis.

|  | active-tDCS (n=43) | sham-tDCS (n=44) | *P*-value |
| --- | --- | --- | --- |
| Age (yr) | 70 (4.4) | 72 (5.4) | 0.090 |
| Gender |  |  | 0.218 |
| Male | 16 (37.2%) | 11 (25%) |  |
| Female | 27 (62.8%) | 33 (75%) |  |
| Weight (kg) | 65.2 (9.3) | 65.1 (12.5) | 0.969 |
| Height (cm) | 161.2 (6.8) | 160.5 (7.8) | 0.667 |
| BMI (kg m^-2^) | 25.0 (3.1) | 25.1 (3.6) | 0.956 |
| ASA |  |  | 0.069 |
| II | 25 (58.1%) | 17 (38.6%) |  |
| III | 18 (41.9%) | 27 (61.4%) |  |
| Type of operation |  |  | 0.935 |
| TKA | 27 (62.8%) | 28 (63.6%) |  |
| THA | 16 (37.2%) | 16 (36.4%) |  |
| Age-adjusted Charlson Comorbidity Index | 3.0 (2.0, 4.0) | 3.0 (3.0, 4.0) | 0.087 |
| FRAIL |  |  | 0.056 |
| Robust | 2 (4.7%) | 8 (18.2%) |  |
| Prefrail | 17 (39.5%) | 10 (22.7%) |  |
| Frail | 24 (55.8%) | 26 (59.1%) |  |
| Quality of Recovery-15 score | 140.0 (138.0, 143.0) | 139.5 (133.0, 143.0) | 0.441 |
| Numeric Rating Scale score at motion | 2.0 (1.0, 4.0) | 2.0 (1.0, 4.0) | 0.935 |
| Numeric Rating Scale score at rest | 1.0 (0.0, 2.0) | 1.0 (0.0, 2.0) | 0.566 |
| Fatigue severity scale score | 53.0 (47.0, 55.0) | 54 (48.5, 58.0) | 0.050 |
| Duration of surgery (min) | 100.0 (80.0, 120.0) | 95.0 (81.3, 110.0) | 0.443 |
| Duration of anesthesia (min) | 125.0 (105.0, 140.0) | 120.0 (105.0, 140.0) | 0.717 |
| Estimated blood loss (ml) | 50.0 (50.0, 150.0) | 100.0 (50.0, 187.5) | 0.775 |
| Femoral nerve block |  |  | 0.158 |
| Yes | 27 (62.8%) | 21 (47.7%) |  |
| No | 16 (37.2%) | 23 (52.2%) |  |

Data are presented as mean (standard deviation), n (%), or median (interquartile range). tDCS, transcranial direct current stimulation; BMI, body mass index; ASA, American Society of Anesthesiologist rating; THA, total hip arthroplasty; TKA, total knee arthroplasty; FRAIL, Fatigue, Resistance, Ambulation, Illness and Loss of Weight Scale.**Table A.6.** Secondary outcomes in per-protocol analysis.

|  | active-tDCS (n=43) | sham-tDCS (n=44) | Difference (95% CI)† | *P*-value |
| --- | --- | --- | --- | --- |
| **Quality of Recovery-15** |  |  |  |  |
| Treatment-by-time interaction |  |  |  | 0.010 |
| Main effect |  |  |  | <0.001 |
| T0 | 140.0 (138.0, 143.0) | 139.0 (133.0, 143.0) | 1.0 (-1.0, 3.0) | 0.240 |
| T1 | 117.0 (105.0, 121.0) | 105.0 (98.0, 111.0) | 10.0 (5.0, 14.0) | 0.001 |
| T2 | 123.0 (114.0, 127.0) | 109.0 (101.3, 116.8) | 12.0 (6.0, 16.0) | <0.001 |
| T3 | 137.0 (134.0, 142.0) | 135.0 (127.0, 140.0) | 3.0 (0.0, 7.0) | 0.007 |
| T4 | 145.0 (144.0, 147.0) | 143.0 (137.0, 145.0) | 3.0 (1.0, 5.0) | 0.006 |
| **Physical comfort** |  |  |  |  |
| Treatment-by-time interaction |  |  |  | 0.040 |
| Main effect |  |  |  | <0.001 |
| T0 | 47.0 (46.0, 48.0) | 47.0 (45.0, 48.0) | 0.0 (0.0, 1.0) | 0.141 |
| T1 | 37.0 (32.0, 41.0) | 34.0 (29.3, 37.8) | 3.0 (0.0, 6.0) | 0.022 |
| T2 | 41.0 (37.0, 44.0) | 35.0 (31.3, 40.5) | 5.0 (2.0, 8.0) | <0.001 |
| T3 | 46.0 (44.0, 47.0) | 44.0 (42.0, 47.0) | 1.0 (0.0, 2.0) | 0.014 |
| T4 | 47.0 (47.0, 49.0) | 47.0 (44.0, 48.0) | 1.0 (0.0, 2.0) | 0.013 |
| **Emotional state** |  |  |  |  |
| Treatment-by-time interaction |  |  |  | 0.002 |
| Main effect |  |  |  | <0.001 |
| T0 | 37.0 (36.0, 38.0) | 38.0 (35.0, 38.8) | 0.0 (-1.0, 1.0) | 0.509 |
| T1 | 34.0 (31.0, 38.0) | 30.0 (26.0, 32.8) | 4.0 (2.0, 6.0) | <0.001 |
| T2 | 36.0 (34.0, 38.0) | 32.0 (30.0, 34.0) | 4.0 (2.0, 6.0) | <0.001 |
| T3 | 38.0 (36.0, 38.0) | 37.0 (34.0, 38.0) | 1.0 (0.0, 2.0) | 0.007 |
| T4 | 39.0 (38.0, 40.0) | 38.0 (37.0, 39.0) | 1.0 (0.0, 2.0) | 0.006 |
| **Physical independence** |  |  |  |  |
| Treatment-by-time interaction |  |  |  | 0.595* |
| Main effect |  |  |  | 0.010 |
| T0 | 18.0 (17.0, 19.0) | 18.0 (16.0, 18.8) | 1.0 (0.0, 1.0) | 0.088 |
| T1 | 4.0 (4.0, 5.0) | 4.0 (4.0, 5.0) | 0.0 (0.0, 0.0) | 0.220 |
| T2 | 5.0 (4.0, 7.0) | 4.0 (4.0, 6.8) | 0.0 (0.0, 1.0) | 0.033 |
| T3 | 17.0 (15.0, 18.0) | 15.0 (13.0, 17.0) | 1.0 (0.0, 2.0) | 0.034 |
| T4 | 19.0 (19.0, 20.0) | 18.0 (17.0, 19.0) | 1.0 (0.0, 1.0) | 0.001 |
| **Psychological support** |  |  |  |  |
| Treatment-by-time interaction |  |  |  | 0.086* |
| Main effect |  |  |  | 0.047 |
| T0 | 20.0 (20.0, 20.0) | 20.0 (20.0, 20.0) | 0.0 (0.0, 0.0) | 0.052 |
| T1 | 20.0 (20.0, 20.0) | 20.0 (20.0, 20.0) | 0.0 (0.0, 0.0) | 0.165 |
| T2 | 20.0 (20.0, 20.0) | 20.0 (20.0, 20.0) | 0.0 (0.0, 0.0) | 0.178 |
| T3 | 20.0 (20.0, 20.0) | 20.0 (20.0, 20.0) | 0.0 (0.0, 0.0) | 1.000 |
| T4 | 20.0 (20.0, 20.0) | 20.0 (20.0, 20.0) | 0.0 (0.0, 0.0) | 0.323 |
| **Pain** |  |  |  |  |
| Treatment-by-time interaction |  |  |  | 0.082* |
| Main effect |  |  |  | 0.025 |
| T0 | 18.0 (16.0, 19.0) | 18.0 (17.0, 19.0) | 0.0 (-1.0, 0.0) | 0.724 |
| T1 | 19.0 (18.0, 20.0) | 18.0 (16.0, 20.0) | 0.0 (0.0, 1.0) | 0.234 |
| T2 | 18.0 (17.0, 20.0) | 17.0 (15.0, 20.0) | 1.0 (0.0, 2.0) | 0.082 |
| T3 | 19.0 (19.0, 20.0) | 18.0 (17.0, 19.0) | 1.0 (0.0, 1.0) | <0.001 |
| T4 | 20.0 (20.0, 20.0) | 20.0 (19.0, 20.0) | 0.0 (0.0, 1.0) | 0.001 |
| **Numerical rating scale at motion** |  |  |  |  |
| Treatment-by-time interaction |  |  |  | 0.316* |
| Main effect |  |  |  | 0.002 |
| T0 | 2.0 (1.0, 4.0) | 2.0 (1.0, 4.0) | 0.0 (0.0, 0.0) | 0.935 |
| T1 | 1.0 (1.0, 2.0) | 1.0 (1.0, 4.0) | 0.0 (-1.0, 0.0) | 0.011 |
| T2 | 2.0 (1.0, 4.0) | 4.0 (1.0, 5.0) | -1.0 (-1.0, 0.0) | 0.022 |
| T3 | 1.0 (1.0, 3.0) | 1.0 (1.0, 4.0) | 0.0 (-1.0, 0.0) | 0.019 |
| T4 | 0.0 (0.0, 1.0) | 1.0 (0.0, 1.8) | 0.0 (-1.0, 0.0) | 0.029 |
| **Numerical rating scale at rest** |  |  |  |  |
| Treatment-by-time interaction |  |  |  | 0.369* |
| Main effect |  |  |  | 0.001 |
| T0 | 1.0 (0.0, 2.0) | 1.0 (0.0, 2.0) | 0.0 (0.0, 0.0) | 0.566 |
| T1 | 0.0 (0.0, 0.0) | 0.0 (0.0, 2.8) | 0.0 (-1.0, 0.0) | 0.015 |
| T2 | 1.0 (0.0, 3.0) | 2.5 (0.0, 3.0) | -1.0 (-2.0, 0.0) | 0.034 |
| T3 | 1.0 (0.0, 2.0) | 1.0 (1.0, 3.0) | 0.0 (-1.0, 0.0) | 0.060 |
| T4 | 0.0 (0.0, 0.0) | 0.0 (0.0, 1.0) | 0.0 (0.0, 0.0) | 0.052 |
| **Fatigue severity scale** |  |  |  |  |
| Treatment-by-time interaction |  |  |  | 0.009 |
| Main effect |  |  |  | <0.001 |
| T0 | 53.0 (47.0, 55.0) | 54.0 (48.3, 58.0) | -2.0 (-6.0, 0.0) | 0.079 |
| T1 | 48.0 (45.0, 54.0) | 55.0 (52.3, 61.0) | -7.0 (-9.0, -3.0) | <0.001 |
| T2 | 46.0 (44.0, 54.0) | 57.0 (49.3, 63.0) | -9.0 (-12.0, -5.0) | <0.001 |
| Nausea and vomiting | 10 (23.3%) | 14 (31.8%) | -8.5% (-27.2%, 10.2%) | 0.372 |
| Hospital length of stay (d) | 10.0 (8.0, 12.0) | 10.0 (8.0, 13.0) | 0.0 (-1.0, 2.0) | 0.487 |

Data are presented as n (%) or median (interquartile range). tDCS, transcranial direct current stimulation; T0, the day before surgery; T1, the 2nd hour postoperatively; T2, the 1st day postoperatively; T3, the 1st month postoperatively; T4, the 3rd month postoperatively; CI, confidence interval.

*: Because no treatment-by-time interaction was found, no Bonferroni correction was made for assessing treatment effect at each time point. †: active-tDCS - sham-tDCS.**Table A.7.** Comparison of Quality of Recovery-15, Numerical rating scale, and Fatigue severity scale scores between groups of per-protocol analysis in total hip arthroplasty.

|  | active-tDCS  (n=16) | sham-tDCS  (n=16) | Median difference (95% CI)† | *P*-value |
| --- | --- | --- | --- | --- |
| **Quality of Recovery-15** |  |  |  |  |
| Treatment-by-time interaction |  |  |  | 0.001 |
| Main effect |  |  |  | 0.002 |
| T0 | 140.5 (131.8, 143.0) | 139.5 (133.3, 143.0) | 0.0 (-4.0, 5.0) | 0.912 |
| T1 | 116.0 (105.0, 118.0) | 100.0 (92.5, 105.8) | 13.0 (6.0, 20.0) | 0.004 |
| T2 | 123.0 (114.8, 127.8) | 109.0 (101.8, 111.0) | 14.0 (8.0, 22.0) | <0.001 |
| T3 | 140.5 (135.5, 144.5) | 135.5 (124.8, 138.8) | 6.0 (1.0, 13.0) | 0.033 |
| T4 | 146.0 (144.3, 148.0) | 143.0 (135.5, 145.8) | 3.0 (1.0, 8.0) | 0.141 |
| **Physical comfort** |  |  |  |  |
| Treatment-by-time interaction |  |  |  | 0.001 |
| Main effect |  |  |  | <0.001 |
| T0 | 47.5 (46.0, 48.0) | 46.0 (45.0, 47.8) | 1.0 (-1.0, 2.0) | 0.493 |
| T1 | 36.5 (30.5, 40.8) | 30.5 (27.5, 32.8) | 5.5 (1.0, 10.0) | 0.011 |
| T2 | 41.5 (38.3, 44.8) | 33.0 (31.3, 37.5) | 7.0 (4.0, 11.0) | <0.001 |
| T3 | 47.0 (45.0, 47.0) | 44.5 (40.3, 47.0) | 2.0 (0.0, 5.0) | 0.022 |
| T4 | 47.0 (46.3, 49.0) | 47.0 (43.3, 48.8) | 1.0 (-1.0, 3.0) | 0.160 |
| **Emotional state** |  |  |  |  |
| Treatment-by-time interaction |  |  |  | 0.003 |
| Main effect |  |  |  | 0.006 |
| T0 | 37.5 (34.3, 38.0) | 38.0 (35.3, 38.8) | 0.0 (-2.0, 1.0) | 0.598 |
| T1 | 35.0 (30.0, 37.8) | 30.0 (24.5, 32.0) | 5.5 (2.0, 10.0) | 0.004 |
| T2 | 36.0 (34.3, 38.0) | 32.0 (30.0, 33.5) | 5.0 (3.0, 7.0) | 0.003 |
| T3 | 38.0 (37.3, 39.0) | 37.0 (33.5, 38.0) | 2.0 (0.0, 3.0) | 0.037 |
| T4 | 40.0 (38.0, 40.0) | 38.0 (36.3, 39.0) | 1.0 (0.0, 2.0) | 0.148 |
| **Physical independence** |  |  |  |  |
| Treatment-by-time interaction |  |  |  | 0.004 |
| Main effect |  |  |  | 0.057 |
| T0 | 18.0 (17.3, 19.0) | 17.5 (16.0, 18.8) | 1.0 (-1.0, 2.0) | 0.427 |
| T1 | 4.0 (4.0, 5.0) | 4.0 (4.0, 4.0) | 0.0 (0.0, 1.0) | 0.490 |
| T2 | 5.0 (4.0, 6.0) | 4.0 (4.0, 4.8) | 1.0 (0.0, 2.0) | 0.056 |
| T3 | 17.0 (15.3, 18.0) | 14.0 (11.3, 16.8) | 3.0 (0.0, 4.0) | 0.041 |
| T4 | 19.0 (19.0, 20.0) | 18.0 (16.3, 19.0) | 1.0 (0.0, 2.0) | 0.147 |
| **Psychological support** |  |  |  |  |
| Treatment-by-time interaction |  |  |  | 0.340* |
| Main effect |  |  |  | 0.172 |
| T0 | 20.0 (20.0, 20.0) | 20.0 (20.0, 20.0) | 0.0 (0.0, 0.0) | 1.000 |
| T1 | 20.0 (20.0, 20.0) | 20.0 (20.0, 20.0) | 0.0 (0.0, 0.0) | 0.539 |
| T2 | 20.0 (20.0, 20.0) | 20.0 (20.0, 20.0) | 0.0 (0.0, 0.0) | 0.564 |
| T3 | 20.0 (20.0, 20.0) | 20.0 (20.0, 20.0) | 0.0 (0.0, 0.0) | 0.780 |
| T4 | 20.0 (20.0, 20.0) | 20.0 (20.0, 20.0) | 0.0 (0.0, 0.0) | 1.000 |
| **Pain** |  |  |  |  |
| Treatment-by-time interaction |  |  |  | 0.297* |
| Main effect |  |  |  | 0.743 |
| T0 | 18.0 (16.3, 18.8) | 18.0 (17.3, 19.0) | 0.0 (-1.0, 0.0) | 0.341 |
| T1 | 18.5 (17.3, 20.0) | 18.5 (17.3, 20.0) | 0.0 (-2.0, 1.0) | 0.780 |
| T2 | 19.0 (18.0, 20.0) | 18.5 (15.0, 20.0) | 0.0 (-1.0, 2.0) | 0.491 |
| T3 | 19.0 (18.3, 20.0) | 19.0 (18.0, 19.0) | 1.0 (0.0, 1.0) | 0.086 |
| T4 | 20.0 (20.0, 20.0) | 19.5 (18.3, 20.0) | 0.0 (0.0, 1.0) | 0.171 |
| **Numerical rating scale at motion** |  |  |  |  |
| Treatment-by-time interaction |  |  |  | 0.597* |
| Main effect |  |  |  | 0.612 |
| T0 | 3.5 (1.0, 4.0) | 1.0 (1.0, 3.8) | 0.0 (0.0, 2.0) | 0.323 |
| T1 | 1.0 (1.0, 3.0) | 1.0 (1.0, 4.0) | 0.0 (-1.0, 0.0) | 0.642 |
| T2 | 1.0 (1.0, 3.0) | 1.0 (1.0, 4.0) | 0.0 (-1.0, 0.0) | 0.616 |
| T3 | 1.0 (0.0, 3.0) | 1.5 (1.0, 3.8) | -1.0 (-2.0, 0.0) | 0.171 |
| T4 | 0.0 (0.0, 1.0) | 1.0 (0.0, 2.0) | 0.0 (-1.0, 0.0) | 0.323 |
| **Numerical rating scale at rest** |  |  |  |  |
| Treatment-by-time interaction |  |  |  | 0.651* |
| Main effect |  |  |  | 0.766 |
| T0 | 1.5 (0.0, 2.0) | 0.0 (0.0, 2.0) | 0.0 (0.0, 2.0) | 0.468 |
| T1 | 0.0 (0.0, 1.0) | 0.0 (0.0, 2.5) | 0.0 (-1.0, 0.0) | 0.669 |
| T2 | 0.5 (0.0, 1.8) | 0.0 (0.0, 2.8) | 0.0 (-1.0, 0.0) | 0.867 |
| T3 | 1.0 (0.0, 2.8) | 1.0 (0.0, 3.0) | 0.0 (-1.0, 0.0) | 0.491 |
| T4 | 0.0 (0.0, 1.0) | 0.0 (0.0, 1.0) | 0.0 (0.0, 0.0) | 0.780 |
| **Fatigue severity scale** |  |  |  |  |
| Treatment-by-time interaction |  |  |  | 0.005 |
| Main effect |  |  |  | <0.001 |
| T0 | 50.5 (44.3, 55.8) | 52.0 (44.5, 55.0) | 0.0 (-6.0, 5.0) | 0.800 |
| T1 | 48.0 (46.0, 53.5) | 56.0 (52.5, 59.8) | -7.0 (-11.0, -2.0) | 0.001 |
| T2 | 45.0 (40.3, 54.0) | 58.0 (53.5, 62.5) | -11.0 (-17.0, -7.0) | <0.001 |

Data are presented as median (interquartile range). tDCS, transcranial direct current stimulation; T0, the day before surgery; T1, the 2nd hour postoperatively; T2, the 1st day postoperatively; T3, the 1st month postoperatively; T4, the 3rd month postoperatively; CI, confidence interval.

*: Because no treatment-by-time interaction was found, no Bonferroni correction was made for assessing treatment effect at each time point. †: active-tDCS－sham-tDCS.**Table A.8.** Comparison of Quality of Recovery-15, Numerical rating scale, and Fatigue severity scale scores between groups of per-protocol analysis in total knee arthroplasty.

|  | active-tDCS  (n=27) | sham-tDCS  (n=28) | Median difference (95% CI)† | *P*-value |
| --- | --- | --- | --- | --- |
| **Quality of Recovery-15** |  |  |  |  |
| Treatment-by-time interaction |  |  |  | 0.398* |
| Main effect |  |  |  | 0.002 |
| T0 | 139.0 (138.0, 143.0) | 139.5 (131.3, 143.0) | 1.0 (-2.0, 4.0) | 0.427 |
| T1 | 117.0 (106.0, 123.0) | 108.0 (102.0, 112.0) | 8.0 (1.0, 14.0) | 0.015 |
| T2 | 122.0 (108.0, 126.0) | 109.5 (100.5, 121.8) | 8.0 (1.0, 16.0) | 0.027 |
| T3 | 137.0 (134.0, 138.0) | 134.0 (127.3, 140.0) | 2.0 (-2.0, 6.0) | 0.224 |
| T4 | 144.0 (143.0, 147.0) | 143.0 (138.3, 145.0) | 2.5 (1.0, 5.0) | 0.013 |
| **Physical comfort** |  |  |  |  |
| Treatment-by-time interaction |  |  |  | 0.663* |
| Main effect |  |  |  | 0.034 |
| T0 | 47.0 (46.0, 48.0) | 47.0 (44.3, 48.0) | 0.0 (-1.0, 1.0) | 0.711 |
| T1 | 38.0 (34.0, 41.0) | 36.0 (31.3, 40.0) | 2.0 (-2.0, 5.0) | 0.418 |
| T2 | 41.0 (36.0, 44.0) | 36.0 (31.5, 42.0) | 3.0 (-1.0, 7.0) | 0.096 |
| T3 | 46.0 (43.0, 46.0) | 44.0 (42.3, 46.0) | 1.0 (-1.0, 2.0) | 0.339 |
| T4 | 47.0 (47.0, 48.0) | 46.5 (44.3, 47.8) | 1.0 (0.0, 2.0) | 0.055 |
| **Emotional state** |  |  |  |  |
| Treatment-by-time interaction |  |  |  | 0.145* |
| Main effect |  |  |  | 0.001 |
| T0 | 37.0 (37.0, 39.0) | 37.5 (35.0, 38.8) | 0.0 (-1.0, 1.0) | 0.542 |
| T1 | 34.0 (32.0, 38.0) | 30.0 (28.0, 34.0) | 4.0 (1.0, 6.0) | 0.009 |
| T2 | 36.0 (34.0, 38.0) | 32.5 (29.3, 35.8) | 3.0 (1.0, 6.0) | 0.006 |
| T3 | 38.0 (36.0, 38.0) | 36.4 (34.0, 38.0) | 0.0 (0.0, 2.0) | 0.261 |
| T4 | 39.0 (38.0, 40.0) | 38.0 (37.0, 38.8) | 1.0 (0.0, 2.0) | 0.005 |
| **Physical independence** |  |  |  |  |
| Treatment-by-time interaction |  |  |  | 0.960* |
| Main effect |  |  |  | 0.059 |
| T0 | 18.0 (17.0, 19.0) | 18.0 (16.0, 18.8) | 1.0 (0.0, 1.0) | 0.171 |
| T1 | 4.0 (4.0, 6.0) | 4.0 (4.0, 5.0) | 0.0 (0.0, 1.0) | 0.370 |
| T2 | 5.0 (4.0, 8.0) | 4.0 (4.0, 8.0) | 0.0 (0.0, 1.0) | 0.251 |
| T3 | 16.0 (15.0, 18.0) | 15.5 (13.0, 17.0) | 1.0 (-1.0, 2.0) | 0.307 |
| T4 | 19.0 (19.0, 20.0) | 18.0 (17.0, 19.0) | 1.0 (0.0, 1.0) | 0.011 |
| **Psychological support** |  |  |  |  |
| Treatment-by-time interaction |  |  |  | 0.141* |
| Main effect |  |  |  | 0.143 |
| T0 | 20.0 (20.0, 20.0) | 20.0 (20.0, 20.0) | 0.0 (0.0, 0.0) | 0.048 |
| T1 | 20.0 (20.0, 20.0) | 20.0 (20.0, 20.0) | 0.0 (0.0, 0.0) | 0.383 |
| T2 | 20.0 (20.0, 20.0) | 20.0 (20.0, 20.0) | 0.0 (0.0, 0.0) | 0.578 |
| T3 | 20.0 (20.0, 20.0) | 20.0 (20.0, 20.0) | 0.0 (0.0, 0.0) | 0.326 |
| T4 | 20.0 (20.0, 20.0) | 20.0 (20.0, 20.0) | 0.0 (0.0, 0.0) | 0.326 |
| **Pain** |  |  |  |  |
| Treatment-by-time interaction |  |  |  | 0.255* |
| Main effect |  |  |  | 0.005 |
| T0 | 18.0 (16.0, 19.0) | 17.5 (16.3, 19.0) | 0.0 (-1.0, 1.0) | 0.757 |
| T1 | 19.0 (18.0, 20.0) | 18.0 (15.3, 19.8) | 1.0 (0.0, 2.0) | 0.080 |
| T2 | 18.0 (17.0, 20.0) | 16.5 (15.0, 19.8) | 1.0 (0.0, 2.0) | 0.063 |
| T3 | 19.0 (19.0, 20.0) | 18.0 (17.0, 19.0) | 1.0 (0.0, 1.0) | 0.003 |
| T4 | 20.0 (20.0, 20.0) | 20.0 (19.0, 20.0) | 0.0 (0.0, 1.0) | 0.003 |
| **Numerical rating scale at motion** |  |  |  |  |
| Treatment-by-time interaction |  |  |  | 0.638* |
| Main effect |  |  |  | <0.001 |
| T0 | 2.0 (1.0, 4.0) | 3.0 (1.0, 4.0) | 0.0 (-1.0, 0.0) | 0.354 |
| T1 | 1.0 (1.0, 1.0) | 1.0 (1.0, 4.0) | -1.0 (-3.0, 0.0) | 0.007 |
| T2 | 3.0 (1.0, 4.0) | 4.0 (3.0, 5.0) | -1.0 (-2.0, 0.0) | 0.013 |
| T3 | 1.0 (1.0, 3.0) | 1.0 (1.0, 4.0) | 0.0 (-1.0, 0.0) | 0.056 |
| T4 | 0.0 (0.0, 1.0) | 1.0 (0.0, 1.0) | 0.0 (-1.0, 0.0) | 0.064 |
| **Numerical rating scale at rest** |  |  |  |  |
| Treatment-by-time interaction |  |  |  | 0.718* |
| Main effect |  |  |  | <0.001 |
| T0 | 0.0 (0.0, 2.0) | 1.5 (0.0, 3.0) | 0.0 (-1.0, 0.0) | 0.166 |
| T1 | 0.0 (0.0, 0.0) | 0.0 (0.0, 2.8) | 0.0 (-2.0, 0.0) | 0.007 |
| T2 | 2.0 (0.0, 3.0) | 3.0 (2.0, 3.8) | -1.0 (-2.0, 0.0) | 0.015 |
| T3 | 1.0 (0.0, 2.0) | 1.0 (1.0, 3.0) | -1.0 (-1.0, 0.0) | 0.073 |
| T4 | 0.0 (0.0, 0.0) | 0.0 (0.0, 1.0) | 0.0 (0.0, 0.0) | 0.022 |
| **Fatigue severity scale** |  |  |  |  |
| Treatment-by-time interaction |  |  |  | 0.141* |
| Main effect |  |  |  | 0.001 |
| T0 | 53.0 (48.0, 55.0) | 55.5 (51.8, 60.0) | -4.0 (-7.0, -1.0) | 0.019 |
| T1 | 49.0 (44.0, 54.0) | 55.0 (50.8, 61.0) | -7.0 (-10.0, -2.0) | 0.001 |
| T2 | 47.0 (45.0, 54.0) | 56.5 (46.0, 63.0) | -8.0 (-11.0, -2.0) | 0.006 |

Data are presented as median (interquartile range). tDCS, transcranial direct current stimulation; T0, the day before surgery; T1, the 2nd hour postoperatively; T2, the 1st day postoperatively; T3, the 1st month postoperatively; T4, the 3rd month postoperatively; CI, confidence interval.

*: Because no treatment-by-time interaction was found, no Bonferroni correction was made for assessing treatment effect at each time point. †: active-tDCS－sham-tDCS.**Table A.9.** Detailed results of the GEE model in the interaction model of intention-to-treat analysis.

|  | active-tDCS (n=48)* | sham-tDCS (n=48)* | Difference (95% CI)† | Wald Chi-Square‡ | *P*-value§ | *P*-value\|\| |
| --- | --- | --- | --- | --- | --- | --- |
| **Quality of Recovery-15** |  |  |  |  | <0.001¶ |  |
| T0 | 137.92 (0.96) | 136.25 (1.20) | 1.67 (-1.33, 4.67) | 1.185 | 0.276 | 0.460 |
| T1 | 114.69 (1.59) | 103.40 (2.04) | 11.29 (6.23, 16.35) | 19.106 | <0.001 | <0.001 |
| T2 | 120.06 (1.64) | 107.13 (2.05) | 12.94 (7.79, 18.08) | 24.288 | <0.001 | <0.001 |
| T3 | 136.91 (0.94) | 130.20 (1.79) | 6.71 (2.75, 10.67) | 11.020 | 0.001 | 0.011 |
| T4 | 144.00 (0.80) | 138.89 (1.30) | 5.10 (2.12, 8.08) | 11.274 | 0.001 | <0.001 |
| **Physical comfort** |  |  |  |  | 0.001¶ |  |
| T0 | 46.46 (0.29) | 45.83 (0.37) | 0.63 (-0.30, 1.55) | 1.742 | 0.187 | 0.266 |
| T1 | 37.23 (0.83) | 32.69 (1.11) | 4.54 (1.81, 7.27) | 10.644 | 0.001 | 0.003 |
| T2 | 40.58 (0.78) | 34.63 (0.99) | 5.96 (3.49, 8.43) | 22.404 | <0.001 | <0.001 |
| T3 | 45.35 (0.33) | 43.36 (0.55) | 1.99 (0.74, 3.24) | 9.764 | 0.002 | 0.021 |
| T4 | 47.10 (0.28) | 45.59 (0.42) | 1.51 (0.53, 2.49) | 9.060 | 0.003 | 0.015 |
| **Emotional state** |  |  |  |  | <0.001¶ |  |
| T0 | 36.52 (0.35) | 36.19 (0.43) | 0.33 (-0.75, 1.42) | 0.364 | 0.546 | 0.873 |
| T1 | 33.79 (0.66) | 29.12 (0.84) | 4.67 (2.57, 6.76) | 19.070 | ＜0.001 | <0.001 |
| T2 | 35.29 (0.60) | 30.75 (0.80) | 4.54 (2.59, 6.49) | 20.799 | ＜0.001 | <0.001 |
| T3 | 37.30 (0.27) | 35.08 (0.61) | 2.22 (0.90, 3.54) | 10.854 | 0.001 | 0.007 |
| T4 | 38.64 (0.25) | 36.78 (0.51) | 1.86 (0.75, 2.97) | 10.756 | 0.001 | <0.001 |
| **Physical independence** |  |  |  |  | 0.386¶ |  |
| T0 | 17.81 (0.28) | 17.25 (0.26) | 0.56 (-0.19, 1.31) | 2.168 | 0.141 | 0.047 |
| T1 | 5.44 (0.37) | 5.02 (0.32) | -0.42 (-0.55, 1.38) | 0.721 | 0.396 | 0.062 |
| T2 | 6.31 (0.41) | 5.33 (0.35) | 0.98 (-0.08, 2.04) | 3.282 | 0.070 | 0.008 |
| T3 | 15.68 (0.37) | 14.21 (0.48) | 1.46 (0.28, 2.64) | 5.912 | 0.015 | 0.020 |
| T4 | 18.76 (0.23) | 17.69 (0.27) | 1.07 (0.38, 1.76) | 9.216 | 0.002 | <0.001 |
| **Psychological support** |  |  |  |  | 0.017¶ |  |
| T0 | 19.98 (0.02) | 19.71 (0.10) | 0.27 (0.08, 0.47) | 7.381 | 0.007 | 0.007 |
| T1 | 19.92 (0.04) | 19.29 (0.24) | 0.63 (0.14, 1.11) | 6.448 | 0.011 | 0.059 |
| T2 | 19.94 (0.05) | 19.54 (0.18) | 0.40 (0.04, 0.75) | 4.705 | 0.030 | 0.071 |
| T3 | 19.97 (0.02) | 19.96 (0.04) | 0.00 (-0.09, 0.10) | 0.010 | 0.922 | 1.000 |
| T4 | 19.99 (0.01) | 19.96 (0.04) | 0.03 (-0.05, 0.12) | 0.632 | 0.427 | 0.323 |
| **Pain** |  |  |  |  | 0.057¶ |  |
| T0 | 17.15 (0.25) | 17.23 (0.27) | -0.08 (-0.80, 0.63) | 0.053 | 0.819 | 0.697 |
| T1 | 18.31 (0.29) | 17.33 (0.41) | 0.98 (0.00, 1.96) | 3.855 | 0.050 | 0.173 |
| T2 | 18.00 (0.33) | 16.88 (0.42) | 1.13 (0.09, 2.16) | 4.519 | 0.034 | 0.055 |
| T3 | 18.83 (0.20) | 17.62 (0.30) | 1.21 (0.50, 1.92) | 11.193 | 0.001 | <0.001 |
| T4 | 19.73 (0.14) | 18.90 (0.23) | 0.83 (0.30, 1.35) | 9.661 | 0.002 | <0.001 |
| **Numerical rating scale at motion** |  |  |  |  | 0.077¶ |  |
| T0 | 2.73 (0.24) | 2.73 (0.24) | 0.00 (-0.66, 0.66) | 0.000 | 1.000 | 0.929 |
| T1 | 1.35 (0.20) | 2.48 (0.26) | -1.13 (-1.76, -0.49) | 12.102 | 0.001 | 0.001 |
| T2 | 2.35 (0.23) | 3.38 (0.27) | -1.02 (-1.70, -0.34) | 8.568 | 0.003 | 0.004 |
| T3 | 1.77 (0.22) | 2.69 (0.26) | -0.92 (-1.58, -0.25) | 7.324 | 0.007 | 0.009 |
| T4 | 0.60 (0.16) | 1.38 (0.24) | -0.78 (-1.35, -0.21) | 7.299 | 0.007 | 0.014 |
| **Numerical rating scale at rest** |  |  |  |  | 0.101¶ |  |
| T0 | 1.31 (0.20) | 1.40 (0.20) | -0.08 (-0.64, 0.48) | 0.085 | 0.770 | 0.716 |
| T1 | 0.44 (0.15) | 1.35 (0.24) | -0.92 (-1.46, -0.37) | 10.951 | 0.001 | 0.002 |
| T2 | 1.31 (0.20) | 2.23 (0.24) | -0.92 (-1.53, -0.30) | 8.612 | 0.003 | 0.007 |
| T3 | 1.15 (0.17) | 1.90 (0.22) | -0.75 (-1.30, -0.20) | 7.189 | 0.007 | 0.019 |
| T4 | 0.32 (0.12) | 0.85 (0.19) | -0.53 (-0.96, -0.09) | 5.638 | 0.018 | 0.031 |
| **Fatigue severity scale** |  |  |  |  | <0.001¶ |  |
| T0 | 51.31 (0.95) | 52.75 (0.99) | -1.44 (-4.12, 1.24) | 1.103 | 0.294 | 0.175 |
| T1 | 49.23 (0.94) | 55.96 (0.86) | -6.73 (-9.22, -4.42) | 28.133 | <0.001 | <0.001 |
| T2 | 47.08 (1.08) | 55.19 (1.17) | -8.10 (-11.22, -4.98) | 25.907 | <0.001 | <0.001 |

tDCS, transcranial direct current stimulation; T0, the day before surgery; T1, the 2nd hour postoperatively; T2, the 1st day postoperatively; T3, the 1st month postoperatively; T4, the 3rd month postoperatively; CI, confidence interval.

*: the estimated marginal mean and standard error. **†**: active-tDCS-sham-tDCS; 95% Wald confidence interval for difference. ‡: Each Wald chi-square tests the simple effects of group within each level combination of the other factors shown. §: Bonferroni correction was made for assessing treatment effect at each time point. ||: No Bonferroni correction was made for assessing treatment effect at each time point. ¶: *P*-value of the group and time interaction.**Table A.10.** Detailed results of the GEE model in the model without time interaction of intention-to-treat analysis.

|  | active-tDCS (n=48)* | sham-tDCS (n=48)* | Difference (95% CI)† | Wald Chi-Square‡ | *P*-value§ |
| --- | --- | --- | --- | --- | --- |
| **Quality of Recovery-15** | 130.71 (0.81) | 123.17 (1.23) | 7.54 (4.58, 10.51) | 24.877 | <0.001 |
| **Physical comfort** | 43.34 (0.33) | 40.42 (0.52) | 2.92 (1.72, 4.13) | 22.723 | <0.001 |
| **Emotional state** | 36.31 (0.30) | 33.58 (0.49) | 2.72 (1.61, 3.84) | 22.969 | <0.001 |
| **Physical independence** | 12.80 (0.21) | 11.90 (0.20) | 0.90 (0.33, 1.46) | 9.709 | 0.002 |
| **Psychological support** | 19.96 (0.02) | 19.69 (0.10) | 0.27 (0.07, 0.46) | 6.936 | 0.008 |
| **Pain** | 18.40 (0.18) | 17.59 (0.23) | 0.81 (0.25, 1.37) | 7.990 | 0.005 |
| **Numerical rating scale at motion** | 1.76 (0.11) | 2.53 (0.18) | -0.77 (-1.17, -0.37) | 14.004 | <0.001 |
| **Numerical rating scale at rest** | 0.91 (0.08) | 1.55 (0.15) | -0.64 (-0.97, -0.31) | 14.085 | <0.001 |
| **Fatigue severity scale** | 49.21 (0.80) | 54.63 (0.78) | -5.42 (-7.60, -3.25) | 23.864 | <0.001 |

tDCS, transcranial direct current stimulation; CI, confidence interval.

*: the estimated marginal mean and standard error. **†**: active-tDCS-sham-tDCS; 95% Wald confidence interval for difference. ‡: The Wald chi-square tests the effect of group. §: Pairwise comparison of estimated marginal means based on the original scale of dependent variables.**Table A.11.** Detailed results of the GEE model in the interaction model of intention-to-treat analysis in total hip arthroplasty.

|  | active-tDCS (n=17)* | sham-tDCS (n=18)* | Difference (95% CI)† | Wald Chi-Square‡ | *P*-value§ | *P*-value\|\| |
| --- | --- | --- | --- | --- | --- | --- |
| **Quality of Recovery-15** |  |  |  |  | 0.001¶ |  |
| T0 | 136.71 (2.21) | 137.33 (1.46) | -0.63 (-5.82, 4.57) | 0.056 | 0.813 | 0.782 |
| T1 | 112.41 (2.57) | 100.33 (2.63) | 12.08 (4.87, 19.29) | 10.778 | 0.001 | 0.002 |
| T2 | 120.71 (2.64) | 106.17 (2.27) | 14.54 (7.72, 21.35) | 17.485 | <0.001 | <0.001 |
| T3 | 138.35 (1.94) | 129.61 (2.92) | 8.74 (1.86, 15.62) | 6.202 | 0.013 | 0.019 |
| T4 | 143.66 (1.95) | 138.63 (2.01) | 5.03 (-0.45, 10.51) | 3.242 | 0.072 | 0.017 |
| **Physical comfort** |  |  |  |  | 0.001¶ |  |
| T0 | 46.41 (0.61) | 46.00 (0.47) | 0.41 (-1.09, 1.92) | 0.287 | 0.592 | 0.318 |
| T1 | 36.35 (1.43) | 30.39 (1.45) | 5.96 (1.97, 9.96) | 8.562 | 0.003 | 0.005 |
| T2 | 41.53 (1.17) | 33.83 (1.15) | 7.70 (4.48, 10.91) | 21.995 | <0.001 | <0.001 |
| T3 | 46.01 (0.51) | 42.84 (1.08) | 3.17 (0.83, 5.50) | 7.065 | 0.008 | 0.058 |
| T4 | 47.19 (0.60) | 45.36 (0.84) | 1.83 (-0.19, 3.85) | 3.166 | 0.075 | 0.204 |
| **Emotional state** |  |  |  |  | 0.001¶ |  |
| T0 | 35.94 (0.73) | 36.61 (0.50) | -0.67 (-2.40, 1.06) | 0.578 | 0.447 | 0.636 |
| T1 | 33.53 (1.11) | 28.61 (1.26) | 4.92 (1.63, 8.21) | 8.576 | 0.003 | 0.007 |
| T2 | 35.59 (0.99) | 31.06 (1.03) | 4.53 (1.74, 7.33) | 10.094 | 0.001 | <0.001 |
| T3 | 37.50 (0.57) | 35.10 (0.85) | 2.41 (0.40, 4.41) | 5.536 | 0.019 | 0.017 |
| T4 | 38.40 (0.63) | 36.92 (0.65) | 1.48 (-0.30, 3.27) | 2.668 | 0.102 | 0.011 |
| **Physical independence** |  |  |  |  | 0.002¶ |  |
| T0 | 17.29 (0.66) | 17.33 (0.38) | -0.04 (-1.53, 1.45) | 0.003 | 0.959 | 0.405 |
| T1 | 4.65 (0.20) | 4.72 (0.38) | -0.08 (-0.93, 0.77) | 0.030 | 0.862 | 0.258 |
| T2 | 5.53 (0.34) | 4.44 (0.28) | 1.08 (0.22, 1.95) | 6.071 | 0.014 | 0.01 |
| T3 | 16.16 (0.62) | 13.79 (0.85) | 2.37 (0.30, 4.44) | 5.046 | 0.025 | 0.053 |
| T4 | 18.64 (0.52) | 17.85 (0.43) | 1.06 (-0.25, 2.38) | 2.504 | 0.114 | 0.025 |
| **Psychological support** |  |  |  |  | 0.286¶ |  |
| T0 | 20.00 (<0.01) | 19.89 (0.11) | 0.11 (-0.10, 0.32) | 1.059 | 0.303 | 0.782 |
| T1 | 19.94 (0.06) | 19.00 (0.52) | 0.94 (-0.09, 1.97) | 3.223 | 0.073 | 0.386 |
| T2 | 19.94 (0.06) | 19.50 (0.30) | 0.44 (-0.15, 1.03) | 2.134 | 0.144 | 0.568 |
| T3 | 19.91 (0.07) | 20.01 (0.02) | -0.11 (-0.24, 0.03) | 2.387 | 0.122 | 0.763 |
| T4 | 19.99 (0.01) | 20.01 (0.01) | -0.02 (-0.05, 0.01) | 1.779 | 0.182 | 1.000 |
| **Pain** |  |  |  |  | 0.328¶ |  |
| T0 | 17.06 (0.45) | 17.50 (0.37) | -0.44 (-1.59, 0.70) | 0.570 | 0.450 | 0.525 |
| T1 | 17.94 (0.59) | 17.78 (0.67) | 0.16 (-1.59, 1.91) | 0.033 | 0.855 | 0.782 |
| T2 | 18.18 (0.60) | 17.33 (0.64) | 0.84 (-0.88, 2.56) | 0.925 | 0.336 | 0.386 |
| T3 | 18.78 (0.46) | 17.97 (0.35) | 0.81 (-0.32, 1.94) | 1.987 | 0.159 | 0.058 |
| T4 | 19.46 (0.36) | 18.88 (0.31) | 0.57 (-0.36, 1.51) | 1.457 | 0.227 | 0.118 |
| **Numerical rating scale at motion** |  |  |  |  | 0.461¶ |  |
| T0 | 2.94 (0.43) | 2.44 (0.36) | 0.50 (-0.60, 1.60) | 0.785 | 0.376 | 0.463 |
| T1 | 1.88 (0.38) | 2.33 (0.42) | -0.45 (-1.56, 0.65) | 0.641 | 0.423 | 0.483 |
| T2 | 1.76 (0.34) | 2.39 (0.38) | -0.62 (-1.62, 0.37) | 1.515 | 0.218 | 0.245 |
| T3 | 1.51 (0.40) | 2.39 (0.34) | -0.88 (-1.90, 0.14) | 2.850 | 0.091 | 0.118 |
| T4 | 0.73 (0.34) | 1.25 (0.33) | -0.52 (-1.44, 0.39) | 1.254 | 0.263 | 0.231 |
| **Numerical rating scale at rest** |  |  |  |  | 0.548¶ |  |
| T0 | 1.59 (0.40) | 1.17 (0.31) | 0.42 (-0.57, 1.41) | 0.700 | 0.403 | 0.546 |
| T1 | 0.76 (0.33) | 1.22 (0.41) | -0.46 (-1.48, 0.56) | 0.771 | 0.380 | 0.546 |
| T2 | 0.82 (0.25) | 1.33 (0.34) | -0.51 (-1.34, 0.32) | 1.434 | 0.231 | 0.443 |
| T3 | 1.10 (0.31) | 1.63 (0.32) | -0.53 (-1.39, 0.34) | 1.412 | 0.235 | 0.345 |
| T4 | 0.55 (0.25) | 0.78 (0.26) | -0.23 (-0.93, 0.47) | 0.408 | 0.523 | 0.606 |
| **Fatigue severity scale** |  |  |  |  | 0.001¶ |  |
| T0 | 50.12 (1.77) | 49.56 (1.72) | 0.56 (-4.28, 5.41) | 0.052 | 0.820 | 0.832 |
| T1 | 49.47 (1.41) | 56.06 (1.31) | -6.58 (-10.36, -2.81) | 11.659 | 0.001 | 0.003 |
| T2 | 46.18 (1.65) | 57.50 (1.29) | -11.32 (-15.43, -7.22) | 29.201 | <0.001 | <0.001 |

tDCS, transcranial direct current stimulation; T0, the day before surgery; T1, the 2nd hour postoperatively; T2, the 1st day postoperatively; T3, the 1st month postoperatively; T4, the 3rd month postoperatively; CI, confidence interval.

*: the estimated marginal mean and standard error. **†**: active-tDCS-sham-tDCS; 95% Wald confidence interval for difference. ‡: Each Wald chi-square tests the simple effects of group within each level combination of the other factors shown. §: Bonferroni correction was made for assessing treatment effect at each time point. ||: No Bonferroni correction was made for assessing treatment effect at each time point. ¶: *P*-value of the group and time interaction.**Table A.12.** Detailed results of the GEE model in the model without time interaction of intention-to-treat analysis in total hip arthroplasty.

|  | active-tDCS (n=17)* | sham-tDCS (n=18)* | Difference (95% CI)† | Wald Chi-Square‡ | *P*-value§ |
| --- | --- | --- | --- | --- | --- |
| **Quality of Recovery-15** | 130.37 (1.60) | 122.41 (1.711) | 7.95 (3.36, 12.54) | 11.537 | 0.001 |
| **Physical comfort** | 43.50 (0.58) | 39.68 (0.72) | 3.81 (2.00, 5.63) | 17.010 | <0.001 |
| **Emotional state** | 36.19 (0.58) | 33.66 (0.642) | 2.53 (0.84, 4.23) | 8.603 | 0.003 |
| **Physical independence** | 12.45 (0.28) | 11.57 (0.33) | 0.88 (0.04, 1.72) | 4.183 | 0.041 |
| **Psychological support** | 19.96 (0.03) | 19.68 (0.17) | 0.27 (-0.06, 0.61) | 2.579 | 0.108 |
| **Pain** | 18.28 (0.40) | 17.89 (0.33) | 0.39 (-0.63, 1.41) | 0.567 | 0.451 |
| **Numerical rating scale at motion** | 1.77 (0.24) | 2.16 (0.26) | -0.40 (-1.08, 0.28) | 1.306 | 0.253 |
| **Numerical rating scale at rest** | 0.97 (0.17) | 1.23 (0.24) | -0.26 (-0.84, 0.32) | 0.782 | 0.377 |
| **Fatigue severity scale** | 48.59 (1.07) | 54.37 (1.00) | -5.78 (-8.65, -2.91) | 15.623 | <0.001 |

tDCS, transcranial direct current stimulation; CI, confidence interval.

*: the estimated marginal mean and standard error. **†**: active-tDCS-sham-tDCS; 95% Wald confidence interval for difference. ‡: The Wald chi-square tests the effect of group. §: Pairwise comparison of estimated marginal means based on the original scale of dependent variables.**Table A.13.** Detailed results of the GEE model in the interaction model of intention-to-treat analysis in total knee arthroplasty.

|  | active-tDCS (n=31)* | sham-tDCS (n=30)* | Difference (95% CI)† | Wald Chi-Square‡ | *P*-value§ | *P*-value\|\| |
| --- | --- | --- | --- | --- | --- | --- |
| **Quality of Recovery-15** |  |  |  |  | 0.079¶ |  |
| T0 | 138.58 (0.83) | 135.60 (1.69) | 2.98 (-0.70, 6.67) | 2.514 | 0.113 | 0.483 |
| T1 | 115.94 (1.98) | 105.23 (2.80) | 10.70 (3.98, 17.42) | 9.746 | 0.002 | 0.002 |
| T2 | 119.71 (2.08) | 107.70 (2.99) | 12.01 (4.88, 19.14) | 10.905 | 0.001 | 0.003 |
| T3 | 136.13 (0.97) | 130.53 (2.26) | 5.60 (0.78, 10.42) | 5.185 | 0.023 | 0.102 |
| T4 | 144.17 (0.63) | 139.03 (1.68) | 5.13 (1.62, 8.65) | 8.180 | 0.004 | 0.008 |
| **Physical comfort** |  |  |  |  | 0.196¶ |  |
| T0 | 46.48 (0.30) | 45.73 (0.53) | 0.75 (-0.44, 1.94) | 1.527 | 0.217 | 0.686 |
| T1 | 37.71 (1.01) | 34.07 (1.51) | 3.64 (0.09, 7.20) | 4.041 | 0.044 | 0.112 |
| T2 | 40.06 (1.01) | 35.10 (1.42) | 4.96 (1.56, 8.37) | 8.147 | 0.004 | 0.012 |
| T3 | 44.99 (0.40) | 43.67 (0.59) | 1.32 (-0.08, 2.73) | 3.412 | 0.065 | 0.144 |
| T4 | 47.05 (0.28) | 45.73 (0.45) | 1.32 (0.28, 2.35) | 6.255 | 0.012 | 0.033 |
| **Emotional state** |  |  |  |  | 0.013¶ |  |
| T0 | 36.84 (0.35) | 35.93 (0.62) | 0.91 (-0.48, 2.29) | 1.644 | 0.200 | 0.613 |
| T1 | 33.94 (0.82) | 29.43 (1.11) | 4.50 (1.80, 7.20) | 10.665 | 0.001 | 0.002 |
| T2 | 35.13 (0.75) | 30.57 (1.11) | 4.56 (1.93, 7.19) | 11.560 | 0.001 | 0.001 |
| T3 | 37.19 (0.28) | 35.07 (0.84) | 2.13 (0.39, 3.86) | 5.765 | 0.016 | 0.106 |
| T4 | 38.77 (0.18) | 36.70 (0.71) | 2.07 (0.64, 3.50) | 8.050 | 0.005 | 0.002 |
| **Physical independence** |  |  |  |  | 0.974¶ |  |
| T0 | 18.10 (0.21) | 17.20 (0.36) | 0.90 (0.08, 1.71) | 4.676 | 0.031 | 0.081 |
| T1 | 5.87 (0.55) | 5.20 (0.46) | 0.67 (-0.73, 2.07) | 0.885 | 0.347 | 0.164 |
| T2 | 6.74 (0.60) | 5.87 (0.51) | 0.88 (-0.66, 2.41) | 1.246 | 0.264 | 0.157 |
| T3 | 15.42 (0.45) | 14.47 (0.56) | 0.96 (-0.45, 2.37) | 1.769 | 0.184 | 0.216 |
| T4 | 18.83 (0.22) | 17.77 (0.34) | 1.06 (0.28, 1.85) | 6.998 | 0.008 | 0.008 |
| **Psychological support** |  |  |  |  | 0.095¶ |  |
| T0 | 19.97 (0.03) | 19.60 (0.14) | 0.37 (0.09, 065) | 6.722 | 0.010 | 0.010 |
| T1 | 19.90 (0.05) | 19.47 (0.23) | 0.44 (-0.02, 0.89) | 3.573 | 0.059 | 0.206 |
| T2 | 19.94 (0.06) | 19.57 (0.22) | 0.37 (-0.08, 0.82) | 2.605 | 0.107 | 0.145 |
| T3 | 20.00 (<0.01) | 19.93 (0.07) | 0.07 (-0.06, 0.20) | 1.078 | 0.299 | 0.317 |
| T4 | 20.00 (<0.01) | 19.93 (0.07) | 0.07 (-0.06, 0.20) | 1.063 | 0.302 | 0.317 |
| **Pain** |  |  |  |  | 0.155¶ |  |
| T0 | 17.19 (0.29) | 17.07 (0.36) | 0.13 (-0.78, 1.04) | 0.075 | 0.784 | 0.988 |
| T1 | 18.52 (0.30) | 17.07 (0.51) | 1.45 (0.30, 2.60) | 6.057 | 0.014 | 0.049 |
| T2 | 17.90 (0.38) | 16.60 (0.54) | 1.30 (0.01, 2.60) | 3.890 | 0.049 | 0.047 |
| T3 | 18.85 (0.19) | 17.40 (0.42) | 1.45 (0.55, 2.35) | 9.882 | 0.002 | 0.002 |
| T4 | 19.87 (0.08) | 18.90 (0.31) | 0.97 (0.35, 1.59) | 9.471 | 0.002 | 0.001 |
| **Numerical rating scale at motion** |  |  |  |  | 0.169¶ |  |
| T0 | 2.61 (0.28) | 2.90 (0.32) | -0.29 (-1.12, 0.54) | 0.460 | 0.498 | 0.465 |
| T1 | 1.06 (0.19) | 2.57 (0.33) | -1.50 (-2.26, -0.75) | 15.274 | <0.001 | 0.001 |
| T2 | 2.68 (0.28) | 3.97 (0.31) | -1.29 (-2.11, -0.47) | 9.416 | 0.002 | 0.004 |
| T3 | 1.90 (0.25) | 2.87 (0.36) | -0.96 (-1.82, -0.11) | 4.894 | 0.027 | 0.031 |
| T4 | 0.53 (0.16) | 1.47 (0.33) | -0.93 (-1.65, -0.21) | 6.468 | 0.011 | 0.044 |
| **Numerical rating scale at rest** |  |  |  |  | 0.220¶ |  |
| T0 | 1.16 (0.22) | 1.53 (0.26) | -0.37 (-1.04, 0.30) | 1.190 | 0.275 | 0.327 |
| T1 | 0.26 (0.13) | 1.43 (0.29) | -1.18 (-1.79, -0.56) | 14.080 | <0.001 | 0.001 |
| T2 | 1.58 (0.26) | 2.77 (0.29) | -1.19 (-1.94, -0.43) | 9.386 | 0.002 | 0.005 |
| T3 | 1.17 (0.20) | 2.07 (0.29) | -0.90 (-1.60, -0.20) | 6.316 | 0.012 | 0.029 |
| T4 | 0.20 (0.11) | 0.90 (0.26) | -0.70 (-1.25, -0.15) | 6.148 | 0.013 | 0.025 |
| **Fatigue severity scale** |  |  |  |  | 0.021¶ |  |
| T0 | 51.87 (1.09) | 54.67 (1.04) | -2.70 (-5.65, 0.25) | 3.211 | 0.073 | 0.044 |
| T1 | 49.10 (1.23) | 55.90 (1.12) | -6.80 (-10.06, -3.55) | 16.798 | <0.001 | <0.001 |
| T2 | 47.58 (1.40) | 53.80 (1.65) | -6.22 (-10.46, -1.98) | 8.252 | 0.004 | 0.002 |

tDCS, transcranial direct current stimulation; T0, the day before surgery; T1, the 2nd hour postoperatively; T2, the 1st day postoperatively; T3, the 1st month postoperatively; T4, the 3rd month postoperatively; CI, confidence interval.

*: the estimated marginal mean and standard error. **†**: active-tDCS-sham-tDCS; 95% Wald confidence interval for difference. ‡: Each Wald chi-square tests the simple effects of group within each level combination of the other factors shown. §: Bonferroni correction was made for assessing treatment effect at each time point. ||: No Bonferroni correction was made for assessing treatment effect at each time point. ¶: *P*-value of the group and time interaction.**Table A.14.** Detailed results of the GEE model in the model without time interaction of intention-to-treat analysis in total knee arthroplasty.

|  | active-tDCS (n=31)* | sham-tDCS (n=30)* | Difference (95% CI)† | Wald Chi-Square‡ | *P*-value§ |
| --- | --- | --- | --- | --- | --- |
| **Quality of Recovery-15** | 130.91 (0.90) | 123.62 (1.77) | 7.29 (3.40, 11.17) | 13.533 | <0.001 |
| **Physical comfort** | 43.26 (0.40) | 40.86 (0.70) | 2.40 (0.83, 3.97) | 8.924 | 0.003 |
| **Emotional state** | 36.37 (0.33) | 33.54 (0.68) | 2.83 (1.36, 4.31) | 14.208 | <0.001 |
| **Physical independence** | 12.99 (0.27) | 12.10 (0.25) | 0.89 (0.17, 1.62) | 5.794 | 0.016 |
| **Psychological support** | 19.96 (0.02) | 19.70 (0.12) | 0.26 (0.02, 0.51) | 4.385 | 0.036 |
| **Pain** | 18.47 (0.17) | 17.41 (0.30) | 1.06 (0.40, 1.73) | 9.774 | 0.002 |
| **Numerical rating scale at motion** | 1.76 (0.11) | 2.75 (0.22) | -0.99 (-1.48, -0.51) | 16.106 | <0.001 |
| **Numerical rating scale at rest** | 0.87 (0.08) | 1.74 (0.18) | -0.87 (-1.26, -0.47) | 18.557 | <0.001 |
| **Fatigue severity scale** | 49.55 (1.08) | 54.79 (1.08) | -5.24 (-8.24, -2.24) | 11.753 | 0.001 |

tDCS, transcranial direct current stimulation; CI, confidence interval.

*: the estimated marginal mean and standard error. **†**: active-tDCS-sham-tDCS; 95% Wald confidence interval for difference. ‡: The Wald chi-square tests the effect of group. §: Pairwise comparison of estimated marginal means based on the original scale of dependent variables.**Table A.15.** Detailed results of the GEE model in the interaction model of per-protocol analysis.

|  | active-tDCS (n=43)* | sham-tDCS (n=44)* | Difference (95% CI)† | Wald Chi-Square‡ | *P*-value§ | *P*-value\|\| |
| --- | --- | --- | --- | --- | --- | --- |
| **Quality of Recovery-15** |  |  |  |  | 0.010¶ |  |
| T0 | 139.09(0.82) | 137.55(1.03) | 1.55 (-1.03, 4.13) | 1.382 | 0.240 | 0.441 |
| T1 | 113.98(1.72) | 105.25(1.88) | 8.73 (3.74, 13.72) | 11.741 | 0.001 | <0.001 |
| T2 | 119.37(1.77) | 109.09(1.86) | 10.28 (5.25, 15.31) | 16.061 | <0.001 | <0.001 |
| T3 | 136.91(1.00) | 131.36(1.81) | 5.54 (1.50, 9.59) | 7.213 | 0.007 | 0.030 |
| T4 | 144.05(0.84) | 139.80(1.28) | 4.25 (1.25, 7.25) | 7.698 | 0.006 | 0.001 |
| **Physical comfort** |  |  |  |  | 0.040¶ |  |
| T0 | 46.77(0.26) | 46.09(0.38) | 0.68 (-0.22, 1.58) | 2.165 | 0.141 | 0.272 |
| T1 | 36.77(0.89) | 33.55(1.10) | 3.22 (0.46, 5.98) | 5.233 | 0.022 | 0.034 |
| T2 | 40.26(0.84) | 35.55(0.92) | 4.71 (2.28, 7.14) | 14.383 | <0.001 | 0.001 |
| T3 | 45.30(0.34) | 43.70(0.55) | 1.60 (0.32, 2.88) | 6.009 | 0.014 | 0.066 |
| T4 | 47.14(0.29) | 45.86(0.42) | 1.28 (0.27, 2.28) | 6.208 | 0.013 | 0.034 |
| **Emotional state** |  |  |  |  | 0.002¶ |  |
| T0 | 36.93(0.31) | 36.61(0.37) | 0.32 (-0.62, 1.26) | 0.436 | 0.509 | 0.822 |
| T1 | 33.67(0.72) | 29.66(0.80) | 4.02 (1.90, 6.13) | 13.904 | <0.001 | <0.001 |
| T2 | 35.14(0.66) | 31.39(0.74) | 3.75 (1.81, 5.70) | 14.282 | <0.001 | <0.001 |
| T3 | 37.26(0.29) | 35.41(0.62) | 1.85 (0.50, 3.19) | 7.207 | 0.007 | 0.024 |
| T4 | 38.63(0.27) | 37.05(0.51) | 1.58 (0.46, 2.70) | 7.645 | 0.006 | <0.001 |
| **Physical independence** |  |  |  |  | 0.595¶ |  |
| T0 | 18.02(0.19) | 17.45(0.25) | 0.57 (-0.04, 1.18) | 3.311 | 0.069 | 0.088 |
| T1 | 5.35(0.41) | 5.09(0.35) | 0.26 (-0.80, 1.31) | 0.229 | 0.632 | 0.220 |
| T2 | 6.14(0.44) | 5.36(0.36) | 0.78 (-0.34, 1.89) | 1.850 | 0.174 | 0.033 |
| T3 | 15.74(0.38) | 14.50(0.47) | 1.24 (0.07, 2.42) | 4.283 | 0.038 | 0.034 |
| T4 | 18.81(0.24) | 17.89(0.25) | 0.93 (0.26, 1.60) | 7.378 | 0.007 | 0.001 |
| **Psychological support** |  |  |  |  | 0.086¶ |  |
| T0 | 19.98(0.02) | 19.80(0.09) | 0.18 (0.00, 0.36) | 3.932 | 0.047 | 0.052 |
| T1 | 19.93(0.04) | 19.48(0.230 | 0.45 (-0.01, 0.91) | 3.751 | 0.053 | 0.165 |
| T2 | 19.95(0.05) | 19.80(0.11) | 0.16 (-0.07, 0.39) | 1.774 | 0.183 | 0.178 |
| T3 | 19.98(0.02) | 19.95(0.05) | 0.02 (-0.08, 0.12) | 0.193 | 0.660 | 1.000 |
| T4 | 20.00(<0.01) | 19.95(0.05) | 0.05 (-0.04, 0.13) | 1.023 | 0.312 | 0.323 |
| **Pain** |  |  |  |  | 0.082¶ |  |
| T0 | 17.40(0.24) | 17.55(0.22) | -0.15 (-0.80, 0.50) | 0.207 | 0.649 | 0.724 |
| T1 | 18.26(0.32) | 17.55(0.38) | 0.71 (-0.26, 1.68) | 2.079 | 0.149 | 0.234 |
| T2 | 17.95(0.36) | 17.00(0.41) | 0.95 (-0.12, 2.02) | 3.049 | 0.081 | 0.082 |
| T3 | 18.86(0.22) | 17.80(0.31) | 1.07 (0.33, 1.80) | 8.123 | 0.004 | <0.001 |
| T4 | 19.72(0.15) | 19.05(0.23) | 0.68 (0.14, 1.21) | 6.196 | 0.013 | 0.001 |
| **Numerical rating scale at motion** |  |  |  |  | 0.316¶ |  |
| T0 | 2.56 (0.25) | 2.57 (0.25) | -0.01 (-0.70, 0.68) | 0.001 | 0.977 | 0.935 |
| T1 | 1.47 (0.21) | 2.32 (0.26) | -0.85 (-1.50, -0.21) | 6.711 | 0.010 | 0.011 |
| T2 | 2.40 (0.24) | 3.20 (0.27) | -0.81 (-1.51, -0.11) | 5.148 | 0.023 | 0.022 |
| T3 | 1.72 (0.22) | 2.50 (0.26) | -0.78 (-1.44, -0.12) | 5.295 | 0.021 | 0.019 |
| T4 | 0.58 (0.16) | 1.20 (0.24) | -0.62 (-1.19, -0.06) | 4.701 | 0.030 | 0.029 |
| **Numerical rating scale at rest** |  |  |  |  | 0.369¶ |  |
| T0 | 1.12 (0.20) | 1.25(0.20) | -0.13 (-0.69, 0.43) | 0.219 | 0.640 | 0.566 |
| T1 | 0.49(0.16) | 1.23(0.23) | -0.74 (-1.30, -0.18) | 6.743 | 0.009 | 0.015 |
| T2 | 1.33(0.20) | 2.09(0.25) | -0.77 (-1.40, -0.13) | 5.596 | 0.018 | 0.034 |
| T3 | 1.16(0.18) | 1.75(0.22) | -0.59 (-1.14, -0.03) | 4.311 | 0.038 | 0.060 |
| T4 | 0.30(0.12) | 0.73(0.19) | -0.42 (-0.86, 0.01) | 3.602 | 0.058 | 0.052 |
| **Fatigue severity scale** |  |  |  |  | 0.009¶ |  |
| T0 | 50.65(1.00) | 53.14(1.00) | -2.49 (-5.26, 0.29) | 3.075 | 0.079 | 0.050 |
| T1 | 48.95(1.01) | 55.43(0.89) | -6.48 (-9.12, -3.84) | 23.096 | <0.001 | <0.001 |
| T2 | 46.86(1.18) | 54.77(1.25) | -7.91 (-11.27, -4.55) | 21.273 | <0.001 | <0.001 |

tDCS, transcranial direct current stimulation; T0, the day before surgery; T1, the 2nd hour postoperatively; T2, the 1st day postoperatively; T3, the 1st month postoperatively; T4, the 3rd month postoperatively; CI, confidence interval.

*: the estimated marginal mean and standard error. **†**: active-tDCS-sham-tDCS; 95% Wald confidence interval for difference. ‡: Each Wald chi-square tests the simple effects of group within each level combination of the other factors shown. §: Bonferroni correction was made for assessing treatment effect at each time point. ||: No Bonferroni correction was made for assessing treatment effect at each time point. ¶: *P*-value of the group and time interaction.**Table A.16.** Detailed results of the GEE model in the model without time interaction of per-protocol analysis.

|  | active-tDCS (n=43)* | sham-tDCS (n=44)* | Difference (95% CI)† | Wald Chi-Square‡ | *P*-value§ |
| --- | --- | --- | --- | --- | --- |
| **Quality of Recovery-15** | 130.68 (0.89) | 124.61 (1.12) | 6.07 (3.28, 8.86) | 18.140 | <0.001 |
| **Physical comfort** | 43.25(0.36) | 40.95(0.48) | 2.30 (1.13, 3.47) | 14.817 | <0.001 |
| **Emotional state** | 36.33(0.33) | 34.02(0.43) | 2.30 (1.24, 3.36) | 18.134 | <0.001 |
| **Physical independence** | 12.81(0.22) | 12.06(0.20) | 0.75 (0.18, 1.33) | 6.617 | 0.010 |
| **Psychological support** | 19.97(0.02) | 19.80(0.09) | 0.17 (0.00, 0.34) | 3.941 | 0.047 |
| **Pain** | 18.44(0.20) | 17.79(0.22) | 0.65 (0.08, 1.22) | 4.999 | 0.025 |
| **Numerical rating scale at motion** | 1.74(0.11) | 2.36(0.16) | -0.61 (-1.00, -0.23) | 9.603 | 0.002 |
| **Numerical rating scale at rest** | 0.88(0.08) | 1.41(0.14) | -0.53 (-0.86, -0.20) | 10.181 | 0.001 |
| **Fatigue severity scale** | 48.82(0.85) | 54.45(0.83) | -5.63 (-7.94, -3.31) | 22.619 | <0.001 |

tDCS, transcranial direct current stimulation; CI, confidence interval.

*: the estimated marginal mean and standard error. **†**: active-tDCS-sham-tDCS; 95% Wald confidence interval for difference. ‡: The Wald chi-square tests the effect of group. §: Pairwise comparison of estimated marginal means based on the original scale of dependent variables.**Table A.17.** Detailed results of the GEE model in the interaction model of per-protocol analysis in total hip arthroplasty.

|  | active-tDCS (n=16)* | sham-tDCS (n=16)* | Difference (95% CI)† | Wald Chi-Square‡ | *P*-value§ | *P*-value\|\| |
| --- | --- | --- | --- | --- | --- | --- |
| **Quality of Recovery-15** |  |  |  |  | 0.001¶ |  |
| T0 | 138.19 (1.78) | 138.44 (1.39) | -0.25 (-4.68, 4.18) | 0.012 | 0.912 | 0.780 |
| T1 | 112.06 (2.71) | 101.00 (2.75) | 11.06 (3.50, 18.63) | 8.212 | 0.004 | 0.004 |
| T2 | 120.94 (2.79) | 107.25 (2.27) | 13.69 (6.64, 20.74) | 14.490 | ＜0.001 | <0.001 |
| T3 | 138.50 (1.99) | 130.75 (3.03) | 7.75 (0.64, 14.86) | 4.561 | 0.033 | 0.029 |
| T4 | 143.75 (1.97) | 139.56 (2.05) | 4.19 (-1.38, 9.76) | 2.170 | 0.141 | 0.023 |
| **Physical comfort** |  |  |  |  | 0.001¶ |  |
| T0 | 46.75 (0.55) | 46.25 (0.48) | 0.50 (-0.93, 1.93) | 0.471 | 0.493 | 0.287 |
| T1 | 36.19 (1.51) | 30.87 (1.44) | 5.31 (1.21, 9.41) | 6.450 | 0.011 | 0.014 |
| T2 | 41.75 (1.22) | 34.38 (1.09) | 7.38 (4.17, 10.58) | 20.334 | ＜0.001 | <0.001 |
| T3 | 46.13 (0.51) | 43.38 (1.09) | 2.75 (0.40, 5.10) | 5.268 | 0.022 | 0.094 |
| T4 | 47.25 (0.60) | 45.81 (0.83) | 1.44 (-0.57, 3.44) | 1.978 | 0.160 | 0.305 |
| **Emotional state** |  |  |  |  | 0.003¶ |  |
| T0 | 36.38 (0.63) | 36.81 (0.54) | -0.44 (-2.06, 1.19) | 0.278 | 0.598 | 0.642 |
| T1 | 33.50 (1.18) | 28.31 (1.39) | 5.19 (1.62, 8.76) | 8.110 | 0.004 | 0.014 |
| T2 | 35.69 (1.05) | 31.19 (1.12) | 4.50 (1.49, 7.51) | 8.613 | 0.003 | <0.001 |
| T3 | 37.56 (0.59) | 35.31 (0.90) | 2.25 (0.14, 4.36) | 4.358 | 0.037 | 0.023 |
| T4 | 38.44 (0.643) | 37.06 (0.70) | 1.38 (-0.49, 3.24) | 2.096 | 0.148 | 0.015 |
| **Physical independence** |  |  |  |  | 0.004¶ |  |
| T0 | 17.88 (0.36) | 17.44 (0.41) | 0.44 (-0.64, 1.52) | 0.631 | 0.427 | 0.381 |
| T1 | 4.50 (0.15) | 4.81 (0.42) | -0.31 (-1.20, 0.57) | 0.477 | 0.490 | 0.423 |
| T2 | 5.44 (0.35) | 4.56 (0.29) | 0.88 (-0.02, 1.77) | 3.638 | 0.056 | 0.035 |
| T3 | 16.13 (0.62) | 13.88 (0.91) | 2.25 (0.09, 4.41) | 4.187 | 0.041 | 0.067 |
| T4 | 18.63 (0.52) | 17.63 (0.45) | 1.00 (-0.35, 2.35) | 2.107 | 0.147 | 0.035 |
| **Psychological support** |  |  |  |  | 0.340¶ |  |
| T0 | 20.00 (<0.01) | 20.00 (<0.01) | 0.00 (0.00, 0.00) | 0.000 | 1.000 | 1.000 |
| T1 | 19.94 (0.06) | 19.19 (0.53) | 0.75 (-0.28, 1.78) | 2.018 | 0.155 | 0.539 |
| T2 | 20.00 (<0.01) | 19.75 (0.17) | 0.25(-0.07, 0.57) | 2.286 | 0.131 | 0.564 |
| T3 | 19.94 (0.06) | 20.00 (<0.01) | -0.06 (-0.18, 0.06) | 1.067 | 0.302 | 0.780 |
| T4 | 20.00 (<0.01) | 20.00 (<0.01) | 0.00 (0.00, 0.00) | 0.000 | 1.000 | 1.000 |
| **Pain** |  |  |  |  | 0.297¶ |  |
| T0 | 17.19 (0.46) | 17.94 (0.24) | -0.75 (-1.77, 0.27) | 2.076 | 0.150 | 0.341 |
| T1 | 17.94 (0.63) | 18.00 (0.64) | -0.06 (-1.82, 1.70) | 0.005 | 0.945 | 0.780 |
| T2 | 18.13 (0.64) | 17.38 (0.70) | 0.75 (-1.10, 2.60) | 0.633 | 0.426 | 0.491 |
| T3 | 18.75 (0.47) | 18.19 (0.35) | 0.56 (-0.58, 1.71) | 0.926 | 0.366 | 0.086 |
| T4 | 19.44 (0.38) | 19.06 (0.30) | 0.38 (-0.56, 1.31) | 0.611 | 0.434 | 0.171 |
| **Numerical rating scale at motion** |  |  |  |  | 0.597¶ |  |
| T0 | 2.88 (0.45) | 2.19 (0.36) | 0.69 (-0.44, 1.81) | 1.437 | 0.231 | 0.323 |
| T1 | 1.94 (0.40) | 2.25 (0.43) | -0.31 (-1.46, 0.84) | 0.284 | 0.594 | 0.642 |
| T2 | 1.88 (0.34) | 2.19 (0.40) | -0.31 (-1.34, 0.71) | 0.356 | 0.551 | 0.616 |
| T3 | 1.56 (0.40) | 2.19 (0.32) | -0.63 (-1.62, 0.37) | 1.507 | 0.220 | 0.171 |
| T4 | 0.75 (0.34) | 1.06 (0.30) | -0.31 (-1.20, 0.57) | 0.481 | 0.488 | 0.323 |
| **Numerical rating scale at rest** |  |  |  |  | 0.651¶ |  |
| T0 | 1.50 (0.42) | 0.94 (0.29) | 0.56 (-0.42, 1.55) | 1.247 | 0.264 | 0.468 |
| T1 | 0.81 (0.35) | 1.19 (0.44) | -0.37 (-1.46, 0.71) | 0.456 | 0.499 | 0.669 |
| T2 | 0.88 (0.26) | 1.13 (0.35) | -0.25 (-1.11, 0.61) | 0.323 | 0.570 | 0.867 |
| T3 | 1.13 (0.31) | 1.44 (0.31) | -0.31 (-1.16, 0.53) | 0.524 | 0.469 | 0.491 |
| T4 | 0.56 (0.25) | 0.63 (0.23) | -0.06 (-0.73, 0.60) | 0.034 | 0.854 | 0.780 |
| **Fatigue severity scale** |  |  |  |  | 0.005¶ |  |
| T0 | 49.44 (1.75) | 50.06 (1.75) | -0.63 (-5.47, 4.22) | 0.064 | 0.800 | 0.838 |
| T1 | 49.00 (1.42) | 55.44 (1.39) | -6.44 (-10.33, -2.54) | 10.484 | 0.001 | 0.005 |
| T2 | 45.94 (1.74) | 57.00 (1.39) | -11.06 (-15.43, -6.70) | 24.650 | <0.001 | <0.001 |

tDCS, transcranial direct current stimulation; T0, the day before surgery; T1, the 2nd hour postoperatively; T2, the 1st day postoperatively; T3, the 1st month postoperatively; T4, the 3rd month postoperatively; CI, confidence interval.

*: the estimated marginal mean and standard error. **†**: active-tDCS-sham-tDCS; 95% Wald confidence interval for difference. ‡: Each Wald chi-square tests the simple effects of group within each level combination of the other factors shown. §: Bonferroni correction was made for assessing treatment effect at each time point. ||: No Bonferroni correction was made for assessing treatment effect at each time point. ¶: *P*-value of the group and time interaction.**Table A.18.** Detailed results of the GEE model in the model without time interaction of per-protocol analysis in total hip arthroplasty.

|  | active-tDCS (n=16)* | sham-tDCS (n=16)* | Difference (95% CI)† | Wald Chi-Square‡ | *P*-value§ |
| --- | --- | --- | --- | --- | --- |
| **Quality of Recovery-15** | 130.69 (1.66) | 123.40 (1.72) | 7.29 (2.61, 11.97) | 9.320 | 0.002 |
| **Physical comfort** | 43.61 (0.61) | 40.14 (0.68) | 3.48 (1.68, 5.27) | 14.396 | <0.001 |
| **Emotional state** | 36.31 (0.60) | 33.74 (0.71) | 2.58 (0.75, 4.40) | 7.672 | 0.006 |
| **Physical independence** | 12.51 (0.28) | 11.66 (0.35) | 0.447 (-0.03, 1.73) | 3.614 | 0.057 |
| **Psychological support** | 19.98 (0.02) | 19.79 (0.14) | 0.19 (-0.08, 0.46) | 1.864 | 0.172 |
| **Pain** | 18.29 (0.43) | 18.11 (0.32) | 0.18 (-0.87, 1.22) | 0.107 | 0.743 |
| **Numerical rating scale at motion** | 1.80 (0.24) | 1.97 (0.25) | -0.18 (-0.85, 0.50) | 0.257 | 0.612 |
| **Numerical rating scale at rest** | 0.98 (0.18) | 1.06 (0.24) | -0.09 (-0.66, 0.49) | 0.088 | 0.766 |
| **Fatigue severity scale** | 48.13 (1.03) | 54.17 (1.06) | -6.04 (-8.93, -3.15) | 16.767 | <0.001 |

tDCS, transcranial direct current stimulation; CI, confidence interval.

*: the estimated marginal mean and standard error. **†**: active-tDCS-sham-tDCS; 95% Wald confidence interval for difference. ‡: The Wald chi-square tests the effect of group. §: Pairwise comparison of estimated marginal means based on the original scale of dependent variables.**Table A.19.** Detailed results of the GEE model in the interaction model of per-protocol analysis in total knee arthroplasty.

|  | active-tDCS (n=27)* | sham-tDCS (n=28)* | Difference (95% CI)† | Wald Chi-Square‡ | *P*-value§ | *P*-value\|\| |
| --- | --- | --- | --- | --- | --- | --- |
| **Quality of Recovery-15** |  |  |  |  | 0.398¶ |  |
| T0 | 139.63 (0.73) | 137.04 (1.41) | 2.59 (-0.52, 5.71) | 2.668 | 0.102 | 0.427 |
| T1 | 115.11 (2.20) | 107.68 (2.37) | 7.43 (1.09, 13.77) | 5.280 | 0.022 | 0.015 |
| T2 | 118.44 (2.26) | 110.14 (2.60) | 8.30 (1.55, 15.05) | 5.815 | 0.016 | 0.027 |
| T3 | 135.96 (1.02) | 131.71 (2.25) | 4.25 (-0.59, 9.08) | 2.965 | 0.085 | 0.224 |
| T4 | 144.22 (0.66) | 139.93 (1.63) | 4.29 (0.84, 7.75) | 5.936 | 0.015 | 0.013 |
| **Physical comfort** |  |  |  |  | 0.663¶ |  |
| T0 | 46.78 (0.26) | 46.00 (0.53) | 0.78 (-0.37, 1.93) | 1.754 | 0.185 | 0.711 |
| T1 | 37.11 (1.08) | 35.07 (1.43) | 2.04 (-1.48, 5.56) | 1.290 | 0.256 | 0.418 |
| T2 | 39.37 (1.09) | 36.21 (1.28) | 3.16 (-0.14, 6.45) | 3.527 | 0.060 | 0.096 |
| T3 | 44.81 (0.43) | 43.89 (0.61) | 0.92 (-0.54, 2.38) | 1.532 | 0.216 | 0.339 |
| T4 | 47.07 (0.30) | 45.89 (0.46) | 1.18 (0.10, 2.26) | 4.609 | 0.032 | 0.055 |
| **Emotional state** |  |  |  |  | 0.145¶ |  |
| T0 | 37.26 (0.29) | 36.50 (0.49) | 0.76 (-0.36, 1.88) | 1.759 | 0.185 | 0.542 |
| T1 | 33.78 (0.92) | 30.43 (0.94) | 3.35 (0.78, 5.92) | 6.516 | 0.011 | 0.009 |
| T2 | 34.81 (0.84) | 31.50 (0.98) | 3.31 (0.79, 5.84) | 6.627 | 0.010 | 0.006 |
| T3 | 37.07 (0.30) | 35.46 (0.83) | 1.61 (-0.12, 3.34) | 3.311 | 0.069 | 0.261 |
| T4 | 38.74 (0.19) | 37.04 (0.70) | 1.71 (0.31, 3.10) | 5.726 | 0.017 | 0.005 |
| **Physical independence** |  |  |  |  | 0.960¶ |  |
| T0 | 18.11 (0.22) | 17.46 (0.31) | 0.65 (-0.09, 1.38) | 2.975 | 0.085 | 0.171 |
| T1 | 5.85 (0.63) | 5.25 (0.49) | 0.60 (-0.96, 2.16) | 0.572 | 0.449 | 0.37 |
| T2 | 6.56 (0.66) | 5.82 (0.53) | 0.73 (-0.91, 2.38) | 0.762 | 0.383 | 0.251 |
| T3 | 15.52 (0.47) | 14.86 (0.51) | 0.66 (-0.70, 2.02) | 0.911 | 0.340 | 0.307 |
| T4 | 18.93 (0.21) | 18.04 (0.29) | 0.89 (0.19, 1.59) | 6.262 | 0.012 | 0.011 |
| **Psychological support** |  |  |  |  | 0.141¶ |  |
| T0 | 19.96 (0.04) | 19.68 (0.13) | 0.28 (0.01, 0.56) | 4.187 | 0.041 | 0.048 |
| T1 | 19.93 (0.05) | 19.64 (0.20) | 0.28 (-0.12, 0.68) | 1.938 | 0.164 | 0.383 |
| T2 | 19.93 (0.07) | 19.82 (0.14) | 0.10 (-0.21, 0.42) | 0.423 | 0.516 | 0.578 |
| T3 | 20.00 (<0.01) | 19.93 (0.07) | 0.07 (-0.07, 0.21) | 1.037 | 0.309 | 0.326 |
| T4 | 20.00 (<0.01) | 19.93 (0.07) | 0.07 (-0.07, 0.21) | 1.037 | 0.309 | 0.326 |
| **Pain** |  |  |  |  | 0.255¶ |  |
| T0 | 17.52 (0.27) | 17.32 (0.31) | 0.20 (-0.62, 1.01) | 0.226 | 0.635 | 0.757 |
| T1 | 18.44 (0.34) | 17.29 (0.45) | 1.16 (0.05, 2.27) | 4.164 | 0.041 | 0.080 |
| T2 | 17.85 (0.43) | 16.79 (0.50) | 1.07 (-0.23, 2.37) | 2.587 | 0.108 | 0.063 |
| T3 | 18.93 (0.20) | 17.57 (0.43) | 1.35 (0.42, 2.29) | 8.135 | 0.004 | 0.003 |
| T4 | 19.89 (0.08) | 19.04 (0.31) | 0.85 (0.23, 1.48) | 7.135 | 0.008 | 0.003 |
| **Numerical rating scale at motion** |  |  |  |  | 0.638¶ |  |
| T0 | 2.37 (0.29) | 2.79 (0.33) | -0.42 (-1.28, 0.45) | 0.891 | 0.345 | 0.354 |
| T1 | 1.19 (0.21) | 2.36 (0.32) | -1.17 (-1.92, -0.42) | 9.370 | 0.002 | 0.007 |
| T2 | 2.70 (0.30) | 3.79 (0.31) | -1.08 (-1.92, -0.24) | 6.405 | 0.011 | 0.013 |
| T3 | 1.81 (0.26) | 2.68 (0.35) | -0.86 (-1.73, 0.00) | 3.849 | 0.050 | 0.056 |
| T4 | 0.48 (0.16) | 1.29 (0.33) | -0.80 (-1.52, -0.08) | 4.790 | 0.029 | 0.064 |
| **Numerical rating scale at rest** |  |  |  |  | 0.718¶ |  |
| T0 | 0.89 (0.20) | 1.43 (0.27) | -0.54 (-1.19, 0.11) | 2.665 | 0.103 | 0.166 |
| T1 | 0.30 (0.15) | 1.25 (0.27) | -0.95 (-1.56, -0.35) | 9.629 | 0.002 | 0.007 |
| T2 | 1.59 (0.27) | 2.64 (0.30) | -1.05 (-1.83, -0.27) | 6.923 | 0.009 | 0.015 |
| T3 | 1.19 (0.22) | 1.93 (0.29) | -0.74 (-1.46, -0.03) | 4.146 | 0.042 | 0.073 |
| T4 | 0.15 (0.11) | 0.79 (0.26) | -0.64 (-1.20, -0.07) | 4.916 | 0.027 | 0.022 |
| **Fatigue severity scale** |  |  |  |  | 0.141¶ |  |
| T0 | 51.37 (1.19) | 54.89 (1.10) | -3.52 (-6.68, -0.36) | 4.767 | 0.029 | 0.019 |
| T1 | 48.93 (1.38) | 55.43 (1.15) | -6.50(-10.02, -2.99) | 13.150 | <0.001 | 0.001 |
| T2 | 47.41 (1.56) | 53.50 (1.75) | -6.09(-10.68, -1.51) | 6.779 | 0.009 | 0.006 |

tDCS, transcranial direct current stimulation; T0, the day before surgery; T1, the 2nd hour postoperatively; T2, the 1st day postoperatively; T3, the 1st month postoperatively; T4, the 3rd month postoperatively; CI, confidence interval.

*: the estimated marginal mean and standard error. †: active-tDCS-sham-tDCS; 95% Wald confidence interval for difference. ‡: Each Wald chi-square tests the simple effects of group within each level combination of the other factors shown. §: Bonferroni correction was made for assessing treatment effect at each time point. ||: No Bonferroni correction was made for assessing treatment effect at each time point. ¶: *P*-value of the group and time interaction.**Table A.20.** Detailed results of the GEE model in the model without time interaction of per-protocol analysis in total knee arthroplasty.

|  | active-tDCS (n=27)* | sham-tDCS (n=28)* | Difference (95% CI)† | Wald Chi-Square‡ | *P*-value§ |
| --- | --- | --- | --- | --- | --- |
| **Quality of Recovery-15** | 130.67 (1.01) | 125.30 (1.44) | 5.37 (1.93, 8.82) | 9.338 | 0.002 |
| **Physical comfort** | 43.03 (0.44) | 41.41 (0.62) | 1.62 (0.12, 3.11) | 4.510 | 0.034 |
| **Emotional state** | 36.33 (0.38) | 34.19 (0.54) | 2.15 (0.85, 3.44) | 10.588 | 0.001 |
| **Physical independence** | 12.99 (0.30) | 12.29 (0.23) | 0.71 (-0.03, 1.44) | 3.560 | 0.059 |
| **Psychological support** | 19.96 (0.02) | 19.80 (0.11) | 0.16 (-0.06, 0.38) | 2.145 | 0.143 |
| **Pain** | 18.53 (0.18) | 17.60 (0.28) | 0.93 (0.28, 1.58) | 7.786 | 0.005 |
| **Numerical rating scale at motion** | 1.71 (0.11) | 2.58 (0.20) | -0.87 (-1.32, -0.42) | 14.215 | <0.001 |
| **Numerical rating scale at rest** | 0.82 (0.08) | 1.61 (0.17) | -0.78 (-1.15, -0.42) | 17.432 | <0.001 |
| **Fatigue severity scale** | 49.23 (1.20) | 54.61 (1.15) | -5.37 (-8.62, -2.12) | 10.510 | 0.001 |

tDCS, transcranial direct current stimulation; CI, confidence interval.

*: the estimated marginal mean and standard error. †: active-tDCS-sham-tDCS; 95% Wald confidence interval for difference. ‡: The Wald chi-square tests the effect of group. §: Pairwise comparison of estimated marginal means based on the original scale of dependent variables.

**Figure A.1.** The photo for electrodes placement and instrumentation use.

A and a: the anode electrode over the left dorsolateral prefrontal cortex, which corresponds to the F3 region of the 10-20 electroencephalogram system; B and b: the cathode electrode over the right orbitofrontal area, which corresponds to the Fp2 region of the 10-20 electroencephalogram system; C and c: the apparatus used in this test.

**Figure A.2.** Comparison of NRS scores between groups.


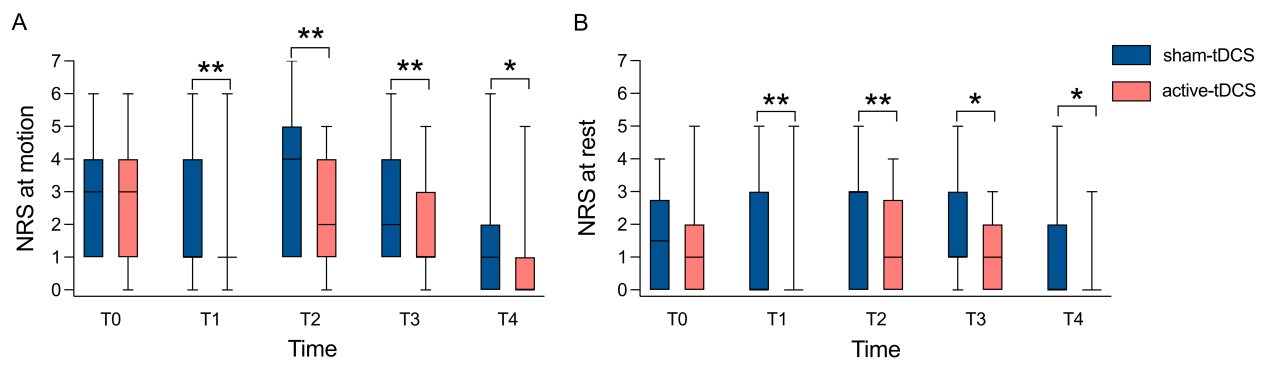


Median values shown as solid line within box of 25th and 75th percentile values. Whiskers represent maximum and minimum values. NRS, numeric rating scale; tDCS, transcranial direct current stimulation. *: *P* < 0.05. **: *P* < 0.01.

**Figure A.3.** The comparison of pain degrees at different time between the active-tDCS and sham-tDCS group.


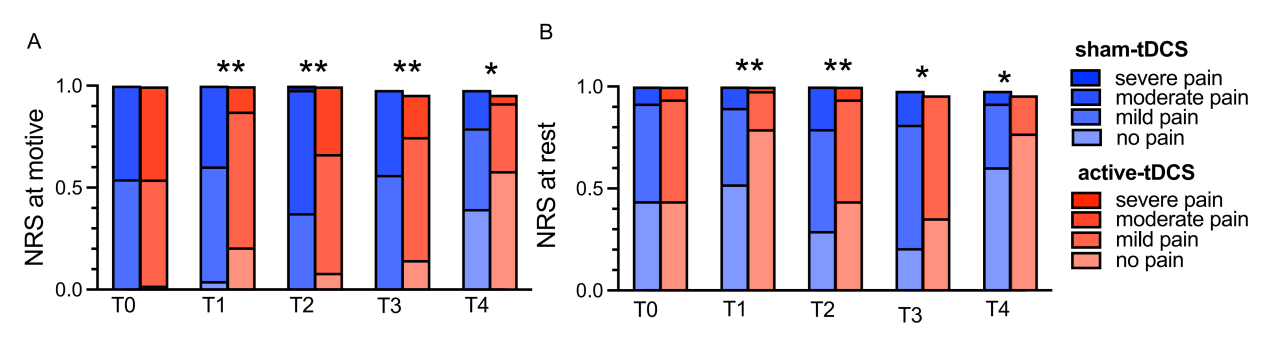


tDCS, transcranial direct current stimulation. *: *P* < 0.05. **: *P* < 0.01.

**Figure A.4.** Comparison of QoR-15 scores and its 5 dimensions between groups in per-protocol analysis.


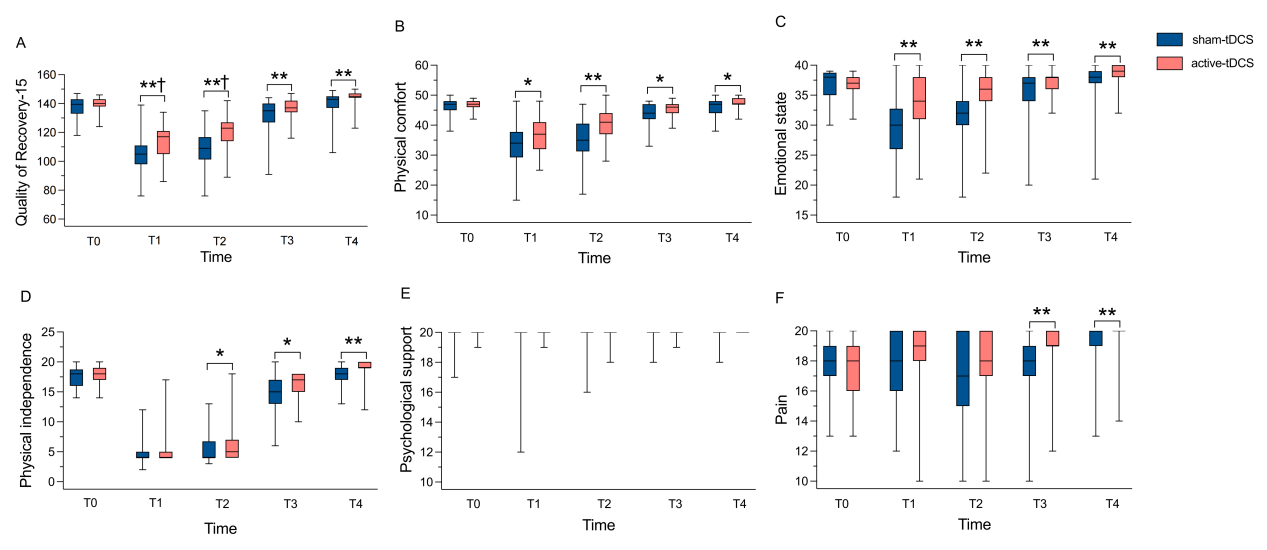


Median values shown as solid line within box of 25th and 75th percentile values. Whiskers represent maximum and minimum values. QoR-15, 15-item quality of recovery; tDCS, transcranial direct current stimulation. *: *P* < 0.05. **: *P* < 0.01. †Median difference > 6.

**Figure A.5.** Comparison of NRS scores between groups in per-protocol analysis.


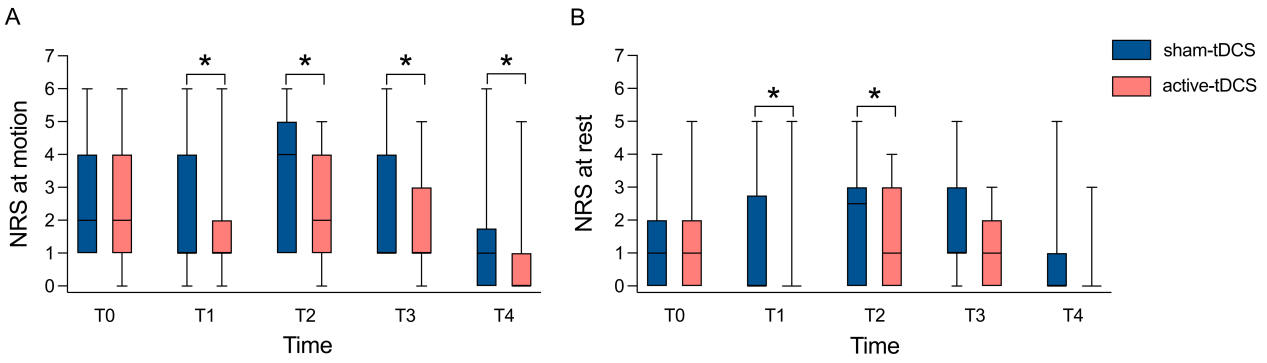


Median values shown as solid line within box of 25th and 75th percentile values. Whiskers represent maximum and minimum values. NRS, numeric rating scale; tDCS, transcranial direct current stimulation. *: *P* < 0.05.

**Figure A.6.** The comparison of pain degrees at different times between groups in per-protocol analysis.


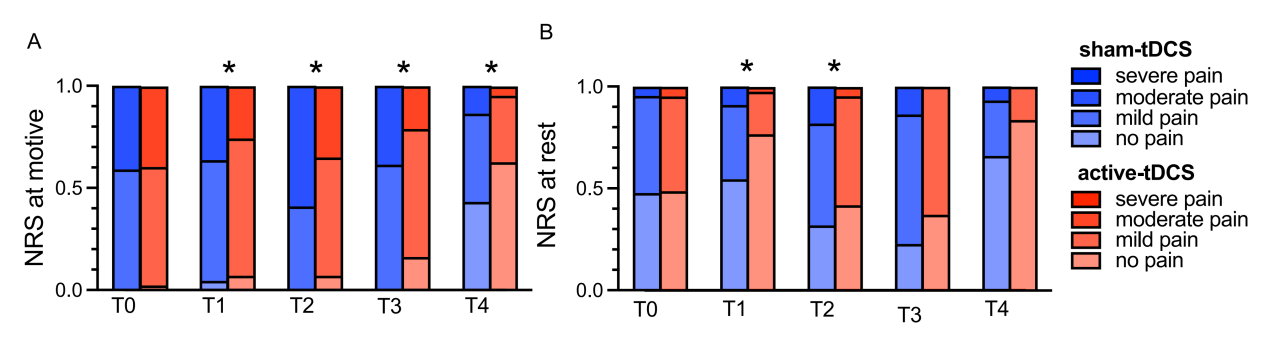


tDCS, transcranial direct current stimulation. *: *P* < 0.05.

**Figure A.7.** Kaplan-Meier survival plot of time to first rescue analgesia in per-protocol analysis.


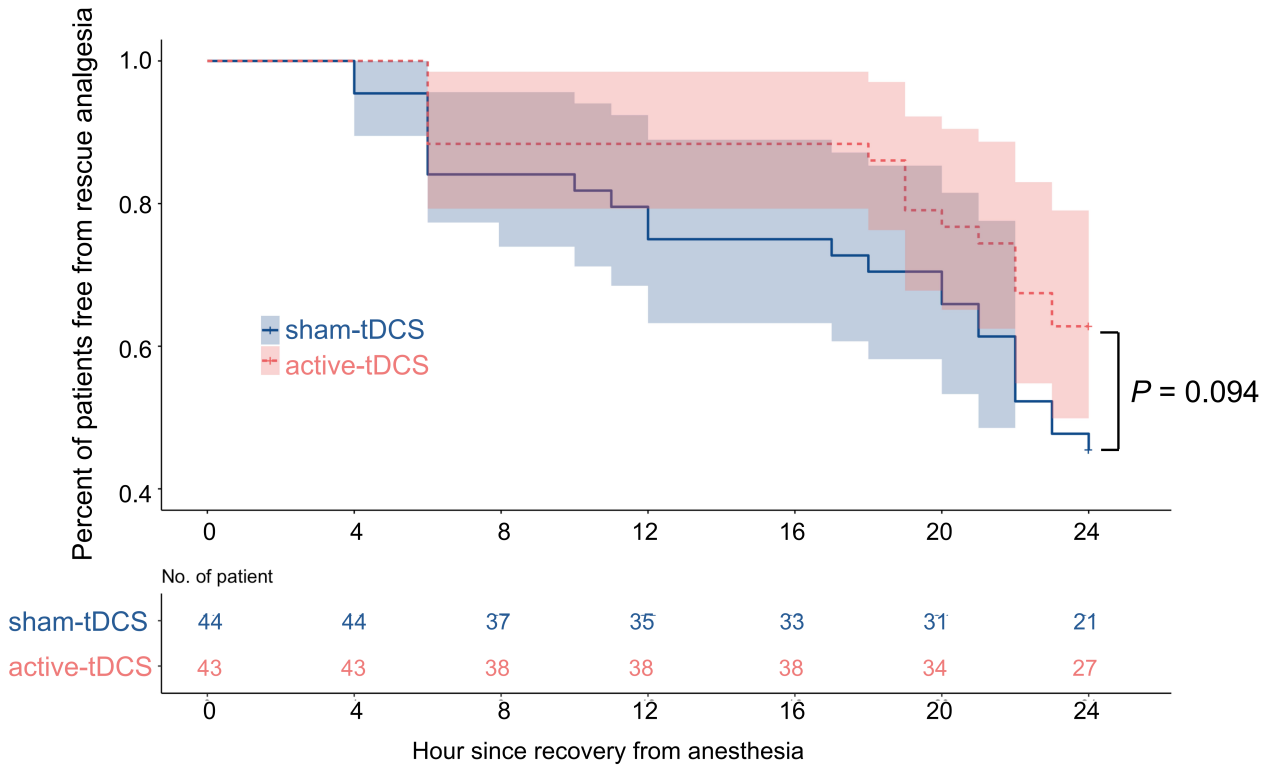


tDCS, transcranial direct current stimulation.
